# Supplementary material for: Single cell RNA-sequencing identified CCR7+/RELB+/IRF1+ T cell responding for juvenile idiopathic arthritis pathogenesis
Source: Front Immunol. 2025 May 8;16:1528446. doi: 10.3389/fimmu.2025.1528446 (PMC12095314; doi:10.3389/fimmu.2025.1528446)
Supplement: Supplementary file 1 [file DataSheet1.pdf]

Supplementary Materials for

# Single cell RNA-sequencing identified CCR7+/RELB+/IRF1+ T cells responding for juvenile idiopathic arthritis pathogenesis

Lewei He[1]#, Xue Gong[1]#, Hui Guo[1]#, Kaiyu Zhou[1], Yue Lan[1], Mingyi Lv[1], Xiaoliang Liu[1], Sha Lin[1], Yimin Hua[1], Junling Guo[2], Zhenxin Fan[1]†, Yifei Li[1]†

1. Key Laboratory of Bioresources and Eco-Environment of MOE, College of Life Sciences, Key Laboratory of Birth Defects and Related Diseases of Women and Children of MOE, Department of Pediatrics, West China Second University Hospital, Sichuan University, Chengdu, Sichuan 610041, China.
2. BMI Center for Biomass Materials and Nanointerfaces, College of Biomass Science and Engineering, Sichuan University, Chengdu, Sichuan 610065, China.
#These authors contributed equally to this work.

**†Correspondence to:**
Zhenxin Fan, PhD, Key Laboratory of Bioresources and Eco-Environment of MOE, College of Life Sciences, Sichuan University, Chengdu, China.
Email: zxfan@scu.edu.cn;

Yifei Li, MD, Key Laboratory of Birth Defects and Related Diseases of Women and Children of MOE, Department of Pediatrics, West China Second University Hospital, Sichuan University, 20 3rd Section, Renmin S.Rd., Chengdu, Sichuan, 610041. Tel: 86-181-8060-1016. Email: liyfwcsh@scu.edu.cn. (Leading contact)

**This PDF file includes:**

Figure S1-S10

**Figure S1**

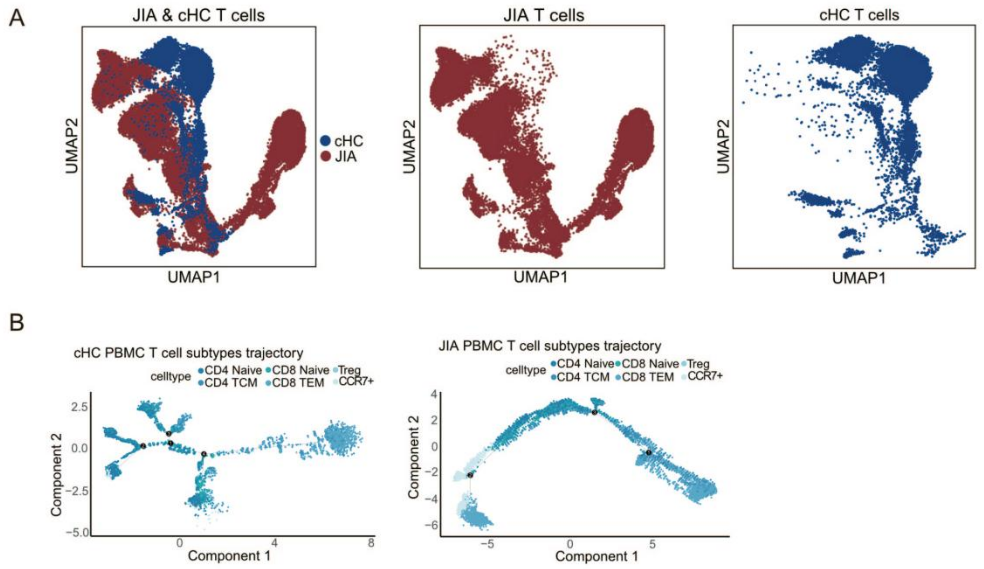


**Figure S1. Distribution and differentiation trajectories of T cells in JIA patients and cHC. (A)** UMAP of high-quality T cells in JIA patients and cHC. **(B)** Differentiation trajectories of T cells in JIA patients and cHC.

**Figure S2**

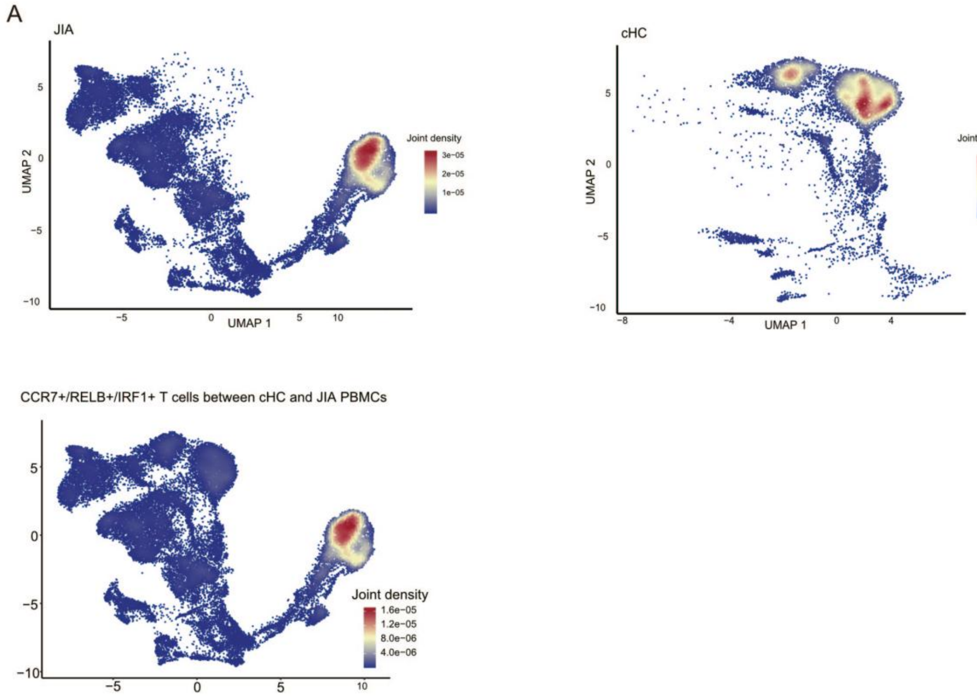


**Figure S2. UMAP projections reflect the gene expression in T cells of JIA patients and cHC. (A)** UMAP projections reflect the expression of *CCR7*, *RELB* and *IRF1* in T cells of JIA patients and cHC.

**Figure S3**

A

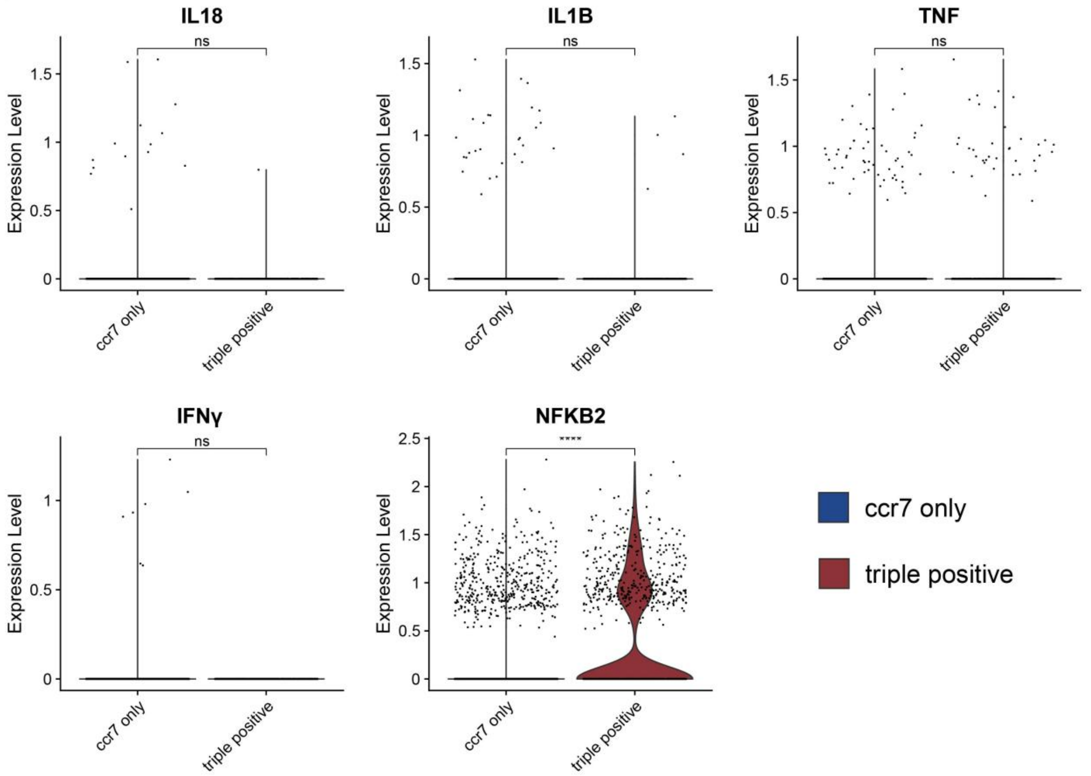


**Figure S3.** Violin plots showing the differences in gene expression levels in CCR7+/RELB+/IRF1+ T cells and CCR7+/RELB-/IRF1- T cells from JIA. **(A)** Violin plots showing the differences in expression levels of IL18, IL1B, TNF, IFNγ and NFKB2 in CCR7+/RELB+/IRF1+ T cells and CCR7+/RELB-/IRF1- T cells from JIA.

**Figure S4**

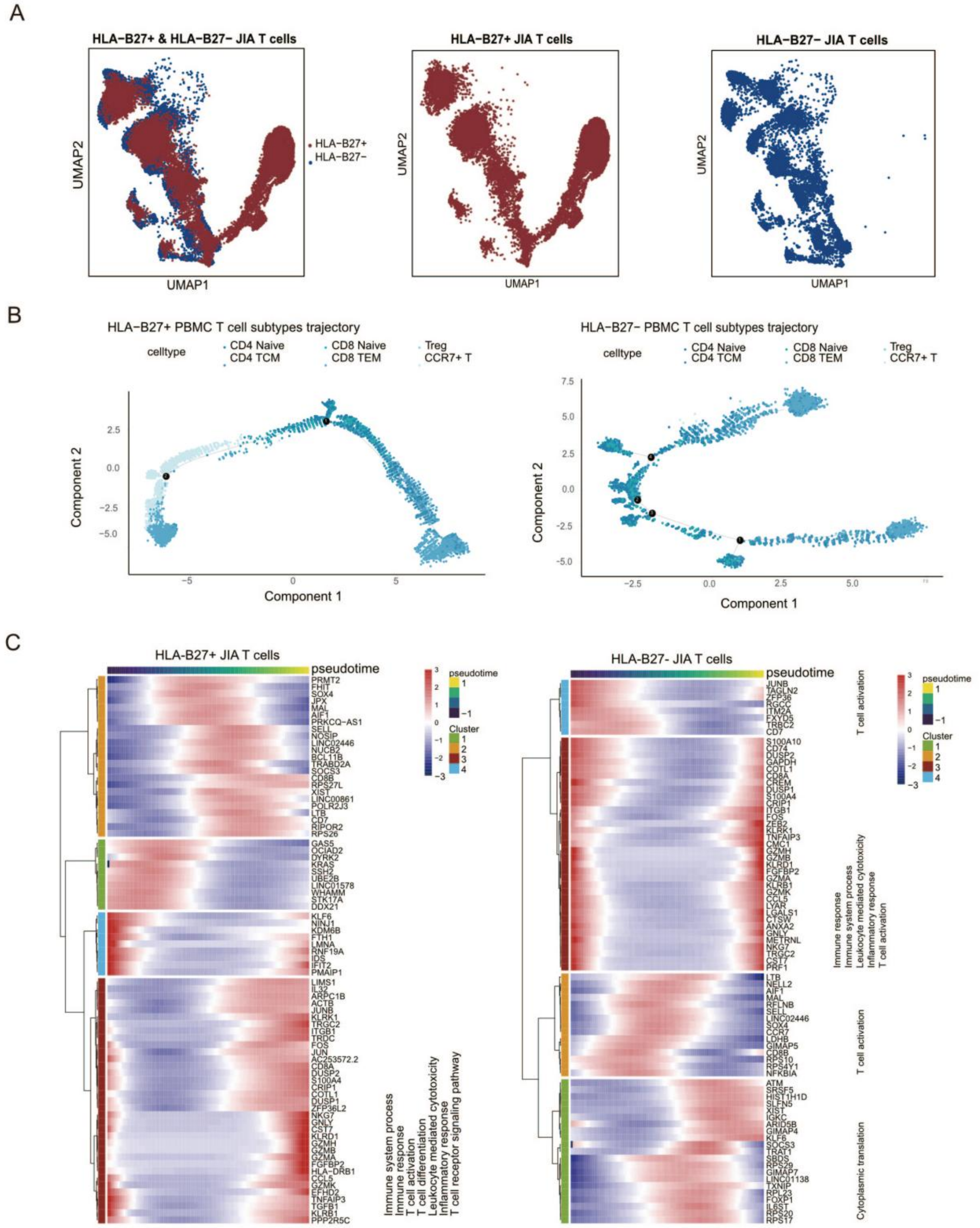


**Figure S4. Distribution and differentiation of T cells in HLA-B27$^{+}$ JIA and HLA-B27$^{-}$ JIA. (A)** UMAP of high-quality T cells in HLA-B27$^{+}$ JIA and HLA-B27$^{-}$ JIA. **(B)** Differentiation trajectories of T cells in HLA-B27$^{+}$ JIA and HLA-B27$^{-}$ JIA. **(C)** Heatmaps showing dynamic changes in gene expression in T cell subtypes of HLA-B27$^{+}$ JIA and HLA-B27$^{-}$ JIA.

**Figure S5**

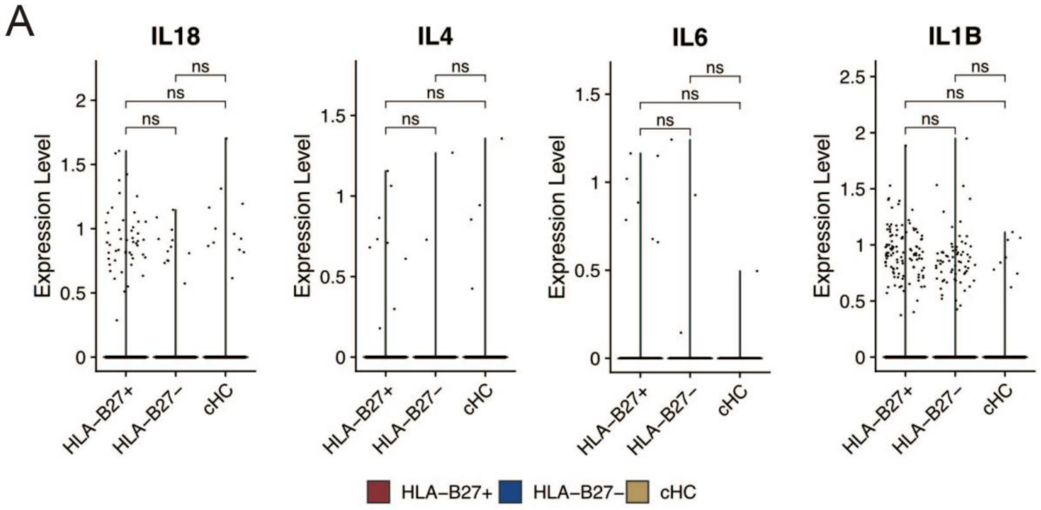


**Figure S5.** Violin plots showing the differences in gene expression levels in T cells from HLA-B27$^{+}$ JIA, HLA-B27$^{-}$ JIA and cHC. **(A)** Violin plots showing the differences in expression levels of IL18, IL4, IL6 and IL1B in T cells from HLA-B27$^{+}$ JIA, HLA-B27$^{-}$ JIA and cHC.

**Figure S6**

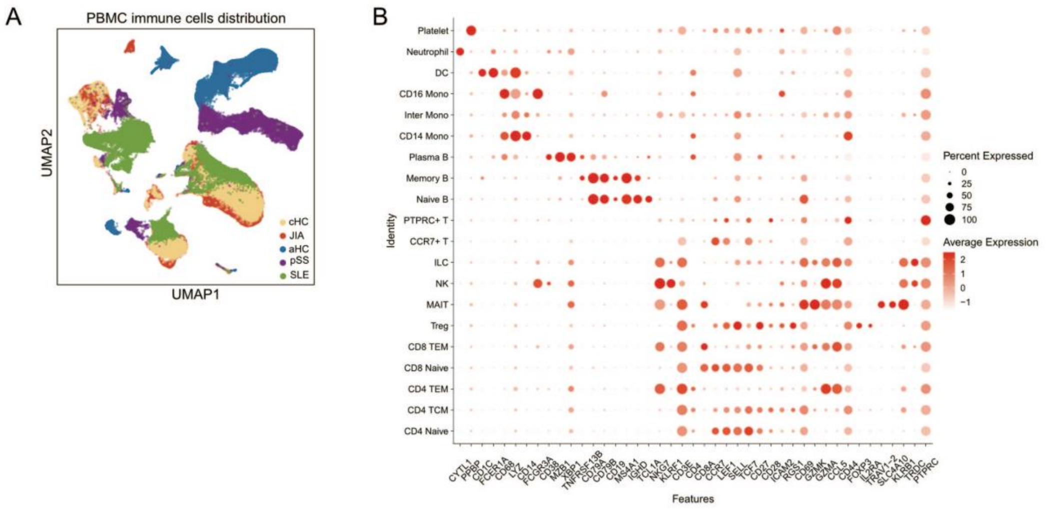


**Figure S6. Immune cells in peripheral blood from JIA patients, cHC, pSS patients, SLE patients and aHC. (A)** UMAP projection of the JIA, cHC, pSS, SLE and aHC. **(B)** Dot plot showing the expression level of classical cell markers used to assign cell identity.

## Figure S7

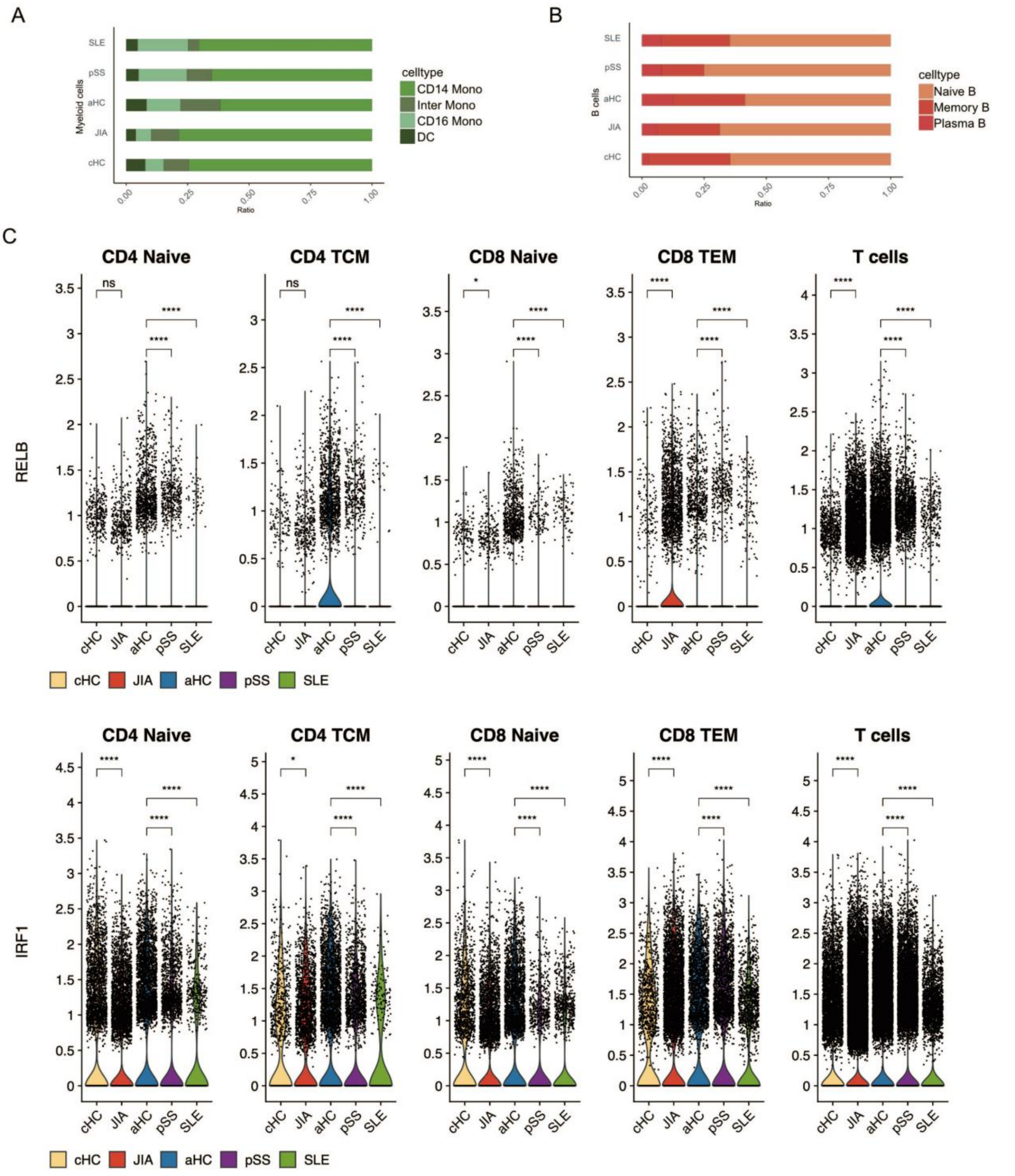


**Figure S7. The proportion of immune cells and gene expression were different among five groups. (A)** The proportion of different subtypes of B cells. **(B)** The proportion of different subtypes of Myeloid cells. **(C)** Violin plots showing the differences in expression levels of RELB and IRF1 in T cell subtypes from cHC, JIA, aHC, pSS and SLE.

**Figure S8**

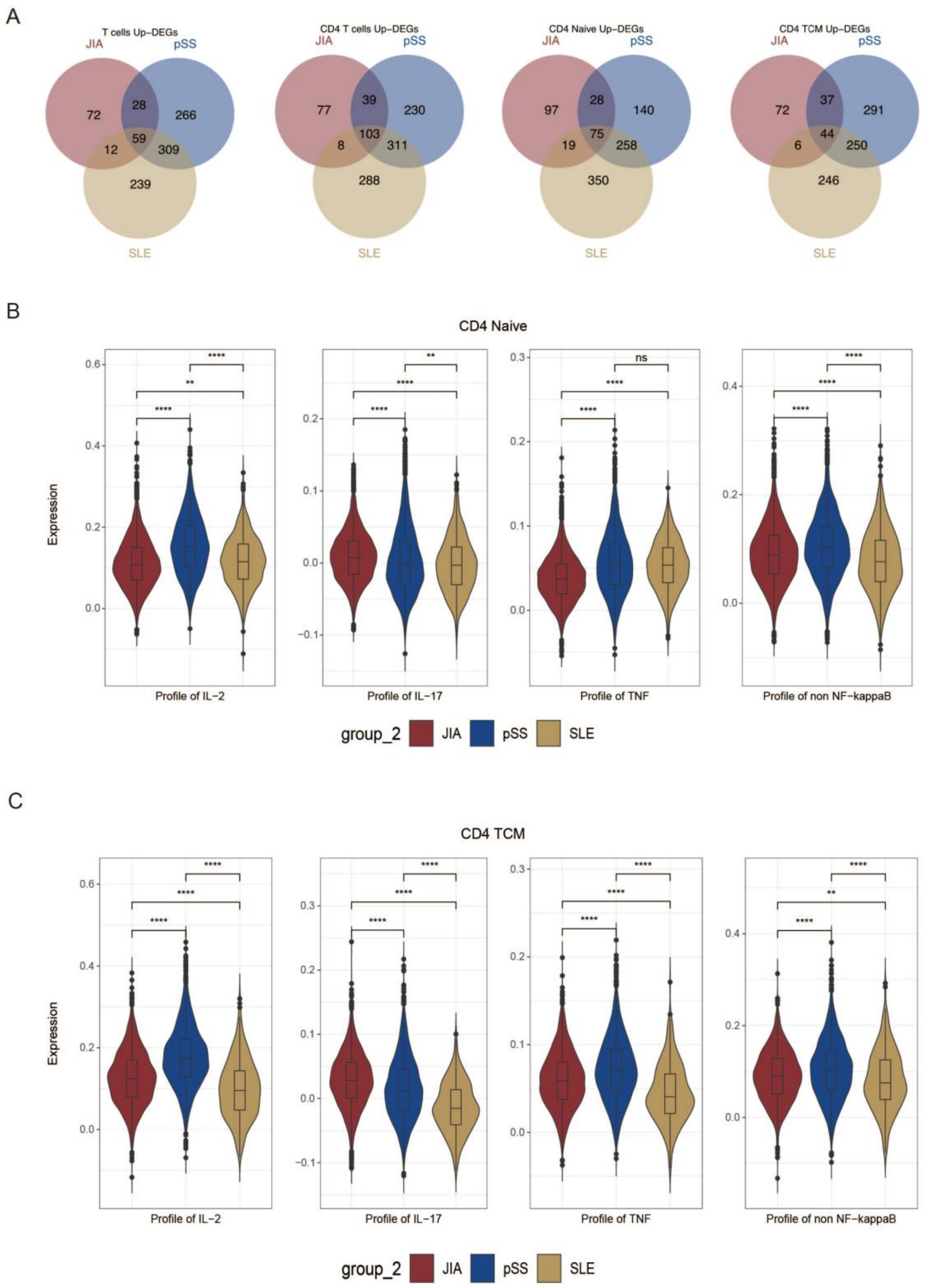


**Figure S8. The DEGs and cytokine expression profile score of T cells and CD4 T cells among the three groups. (A)** Venn plots for the overlap of the upregulated DEGs in T cells and T cell subtypes among three groups. **(B)** Profile scores of IL-2, IL-17, TNF and NF-kappaB in CD4 Naive among three groups. **(C)** Profile scores of IL-2, IL-17, TNF and NF-kappaB in CD4 TCM among three groups.

**Figure S9**

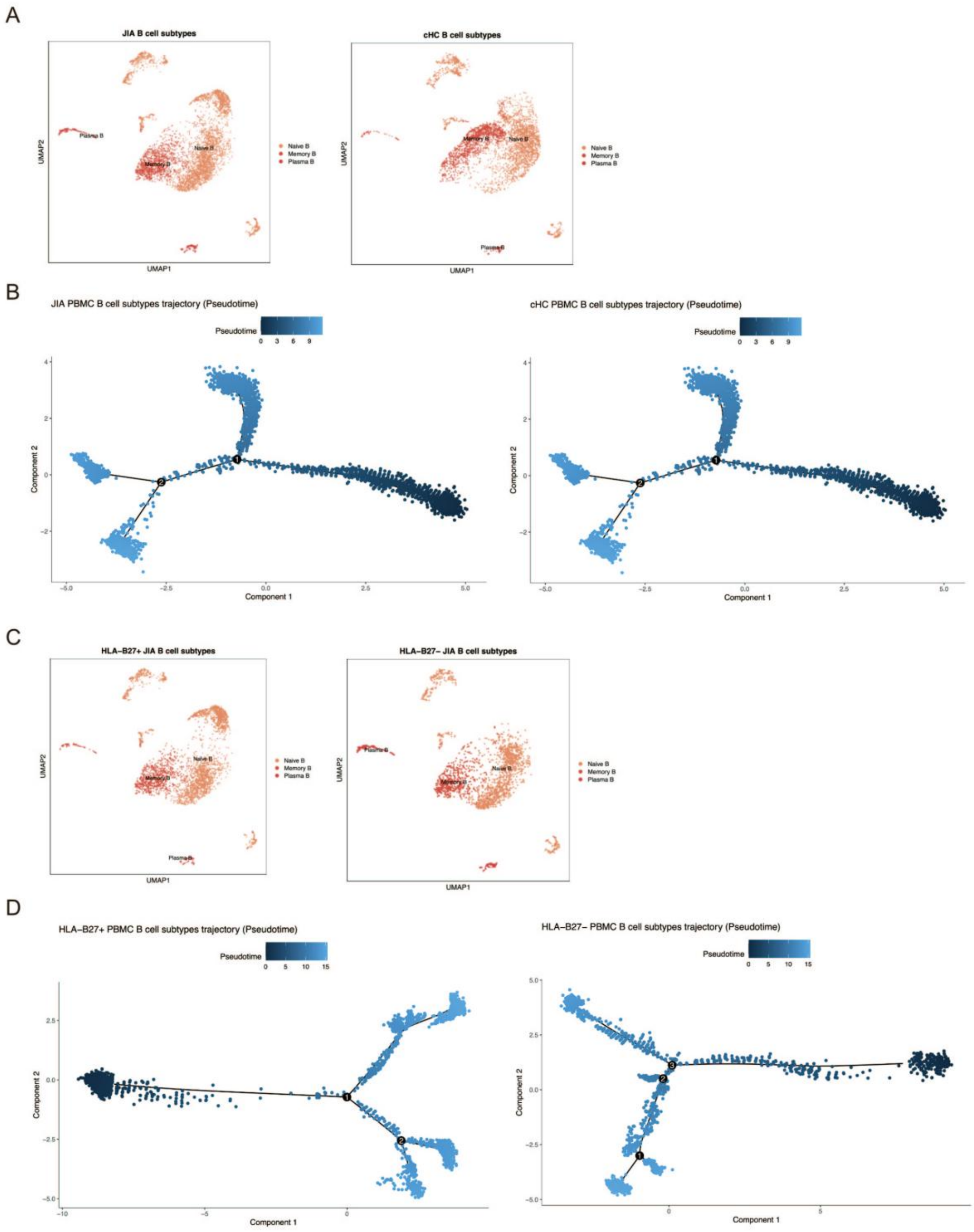


**Figure S9. Classification and differentiation trajectories of B cells in JIA patients. (A)** Integrated UMAP graph of B cell subtypes derived from JIA and cHC. **(B)** Trajectory plots of B cell subtypes in JIA and cHC. **(C)** Integrated UMAP graph of B cell subtypes derived from HLA-B27$^{+}$ JIA and HLA-B27$^{-}$ JIA. **(D)** Trajectory plots of B cell subtypes in HLA-B27$^{+}$ JIA and HLA-B27$^{-}$ JIA.

## Figure S10

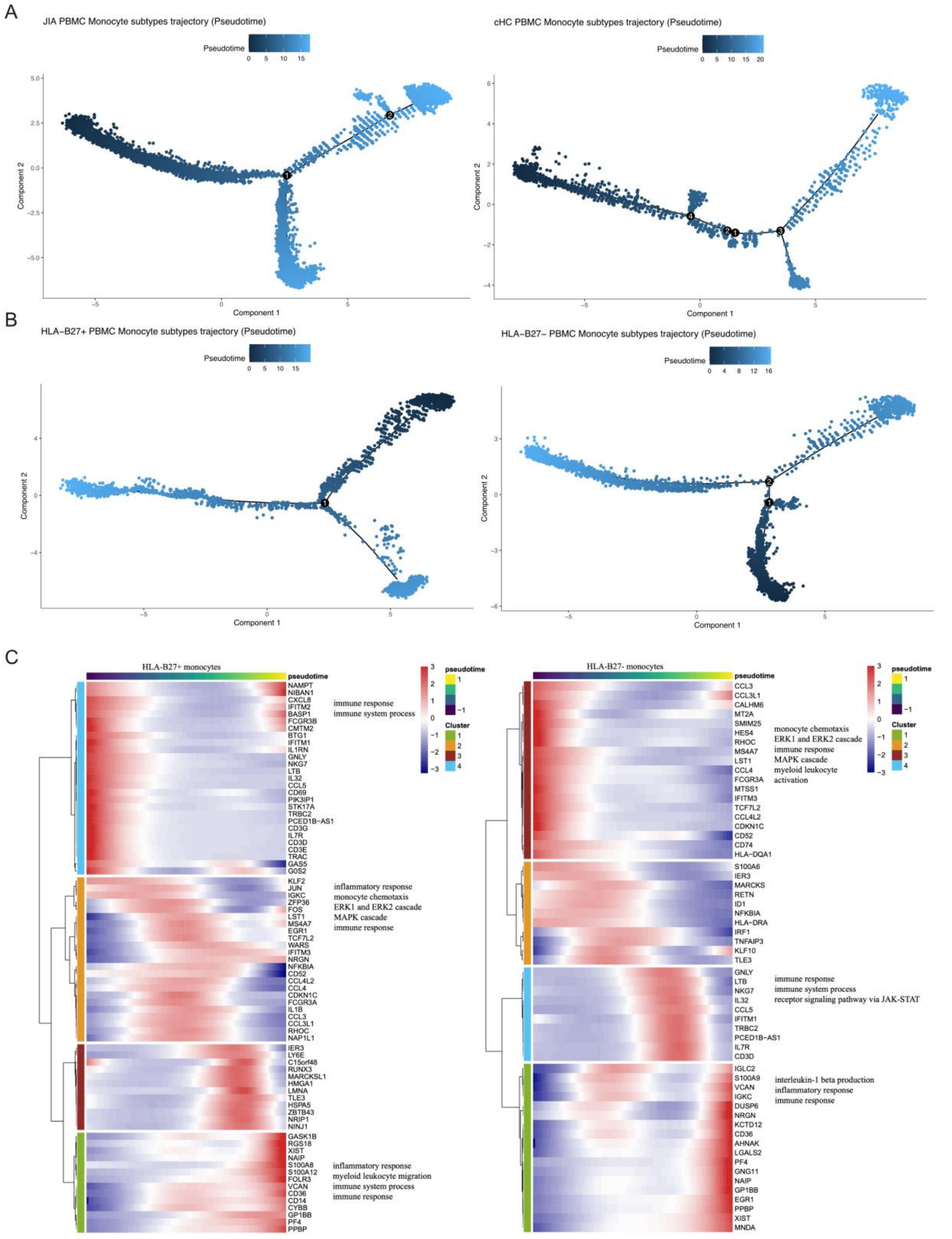


**Figure S10. Differentiation trajectories of Myeloid cells in JIA patients. (A)** Trajectory plots of Monocyte subtypes in JIA and cHC. **(B)** Trajectory plots of Monocyte subtypes in HLA-B27$^{+}$ JIA and HLA-B27$^{-}$ JIA. **(C)** Heatmaps showing dynamic changes in gene expression in Monocyte subtypes of HLA-B27$^{-}$ JIA and HLA-B27$^{+}$ JIA.
