# Supplementary material for: Single cell RNA-sequencing identified CCR7+/RELB+/IRF1+ T cell responding for juvenile idiopathic arthritis pathogenesis
Source: Front Immunol. 2025 May 8;16:1528446. doi: 10.3389/fimmu.2025.1528446 (PMC12095314; doi:10.3389/fimmu.2025.1528446)
Supplement: Supplementary file 2 [file Table1.docx]

Supplementary Table 1. Clinical information of involved cases in the scRNA-seq analysis

|  | **JIA_HLA-B27 Positive** | **JIA_HLA-B27 Negative** | **Healthy Control** |
| --- | --- | --- | --- |
| Gender |  |  |  |
| Female | 2 | 1 | 1 |
| Male | 2 | 2 | 2 |
| HLA-B27 | Positive (+) | Negative (-) | Negative (-) |
| Weight(kg) | 28.45±13.43 | 27.89±9.16 | 23.98±8.78 |
| White blood cell (×10^9^/L) | 12.15±2.92 | 10.03±1.59 | 6.40±1.57 |
| Neutrophils (×10^9^/L) | 7.61±1.72 | 7.13±1.78 | 2.17±0.19 |
| Neutrophils (%) | 63.05±5.93 | 71.20±16.95 | 36.37±10.34 |
| Lymphocyte(×10^9^/L) | 3.73±1.44 | 2.12±1.51 | 3.61±1.89 |
| Lymphocyte (%) | 30.38±7.04 | 21.07±14.33 | 52.90±15.39 |
| Hemoglobin (g/L) | 109.98±14.95 | 127.33±18.79 | 141.00±12.03 |
| Platelet (×10^9^/L) | 430.75±78.60 | 431.33±31.20 | 320.67±19.26 |
| CRP (mg/L) | 46.75±24.78 | 40.55±28.56 | 2.33±1.25 |
| ESR (mm/h) | 67.25±29.87 | 51.00±32.17 | 10.67±5.31 |
| Alanine aminotransferase (ALT) | 26.25±13.44 | 11.67±1.25 | 14.33±1.70 |
| Aspartate aminotransferase (AST) | 29.00±6.16 | 26.00±4.55 | 28.00±3.56 |
| Total Bilirubin (TB, umol/L) | 8.78±7.05 | 9.07±4.27 | 7.13±1.55 |
| Albumin (ALB, g/L) | 37.28±2.21 | 44.47±0.82 | 47.00±1.87 |
| Globulin (GLB, g/L) | 33.18±4.19 | 26.73±4.31 | 30.30±0.43 |
| Urea nitrogen (UN, mmol/L) | 3.09±0.68 | 4.25±0.94 | 3.75±0.25 |
| Creatinine (Cr, umol/L) | 30.25±3.70 | 34.00±12.03 | 34.33±7.36 |
| [Blood](javascript:;) [glucose](javascript:;) (GLu, mmol/L) | 5.52±0.99 | 4.14±0.18 | 4.78±0.33 |
| Immunoglobulin G (IgG, g/L) | 11.16±2.65 | 12.13±0.54 | 12.17±1.26 |
| Immunoglobulin A (IgA, g/L) | 2.40±0.41 | 1.65±0.55 | 1.72±0.35 |
| Immunoglobulin M (IgM, g/L) | 1.56±0.29 | 1.59±0.53 | 2.05±0.60 |
| Complement 3 (C3, g/L) | 1.25±0.35 | 1.26±0.25 | 1.06±0.21 |
| Complement 4 (C4, g/L) | 0.27±0.69 | 0.21±0.10 | 0.19±0.04 |
| α1-acid glycoprotein (α1-AG, g/L) | 2.55±0.75 | 1.05±0.57 | 0.68±0.06 |
| Antistreptohaemolysin O (ASO) (positive cases, n) | 2 | 1 | 0 |
| Rheumatoid factors (RF) (positive cases, n) | 3 | 2 | 0 |
| Anti-cyclic citrullinated peptide antibodies (CCP-IgG) | 1.26±1.42 | 0.45±0.18 | 0.34±0.29 |
| Anti-neutrophil cytoplasmic antibodies (ANCA) (positive cases, n) | 1 | 1 | 0 |
| Macrophage Activation Syndrome (MAS) (positive cases, n) | 0 | 0 | 0 |
| Lesion of Pulmonary (positive cases, n) | 1 | 0 | 0 |
| Destruction of bone (positive cases, n) | 3 | 3 | 0 |
| Joint effusion (positive cases, n) | 2 | 3 | 0 |
| JADAS score pre-treatment | 16.50±3.61 | 17.67±2.28 | - |
