# Supplementary figures and images for: Single cell RNA-sequencing identified CCR7+/RELB+/IRF1+ T cell responding for juvenile idiopathic arthritis pathogenesis
Source: Front Immunol. 2025 May 8;16:1528446. doi: 10.3389/fimmu.2025.1528446 (PMC12095314; doi:10.3389/fimmu.2025.1528446)

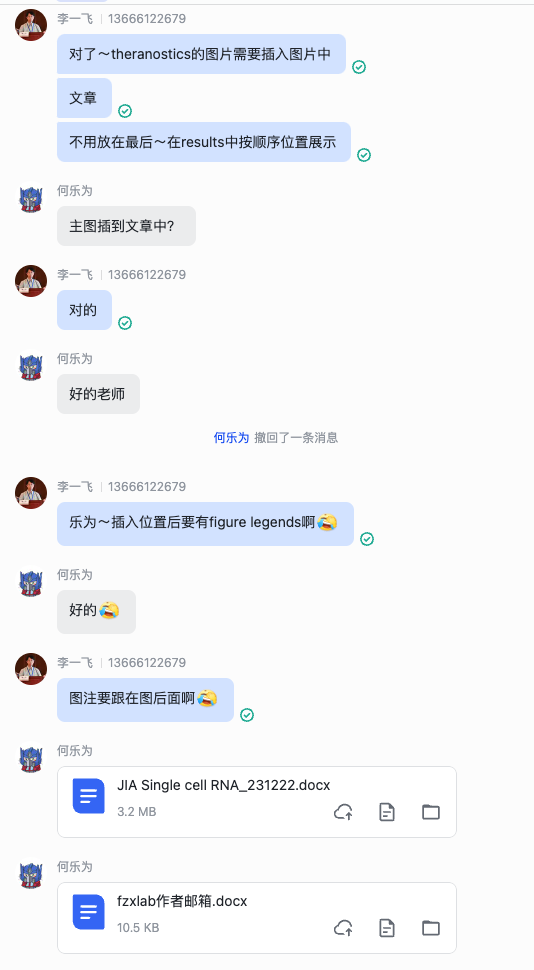

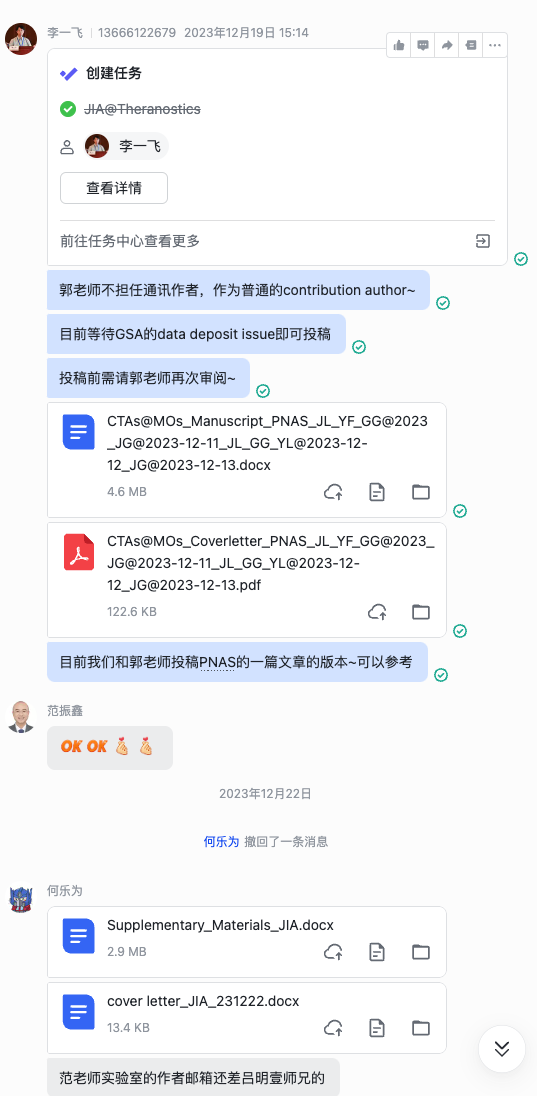


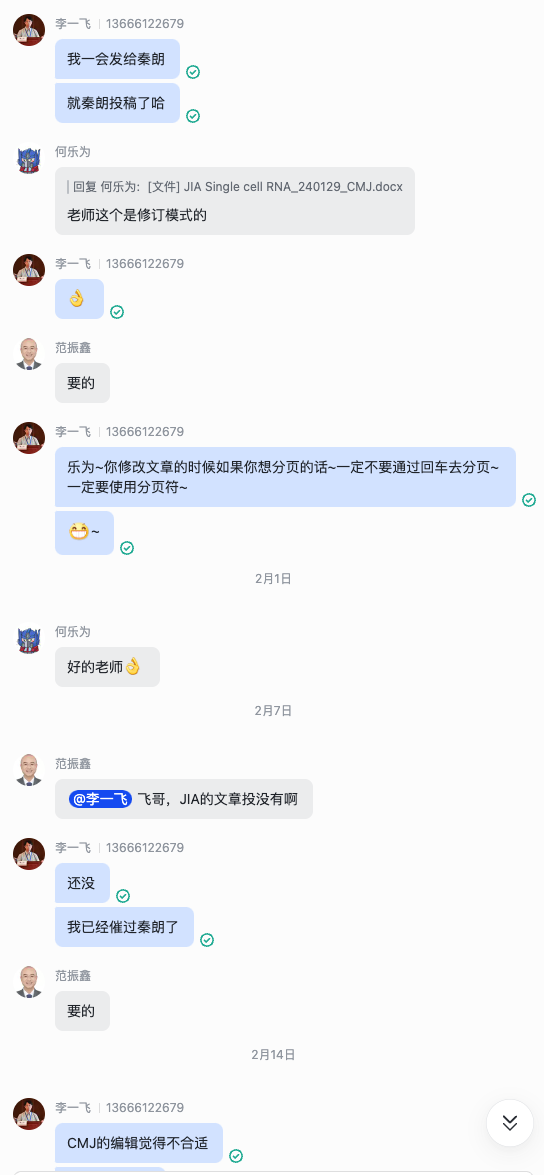


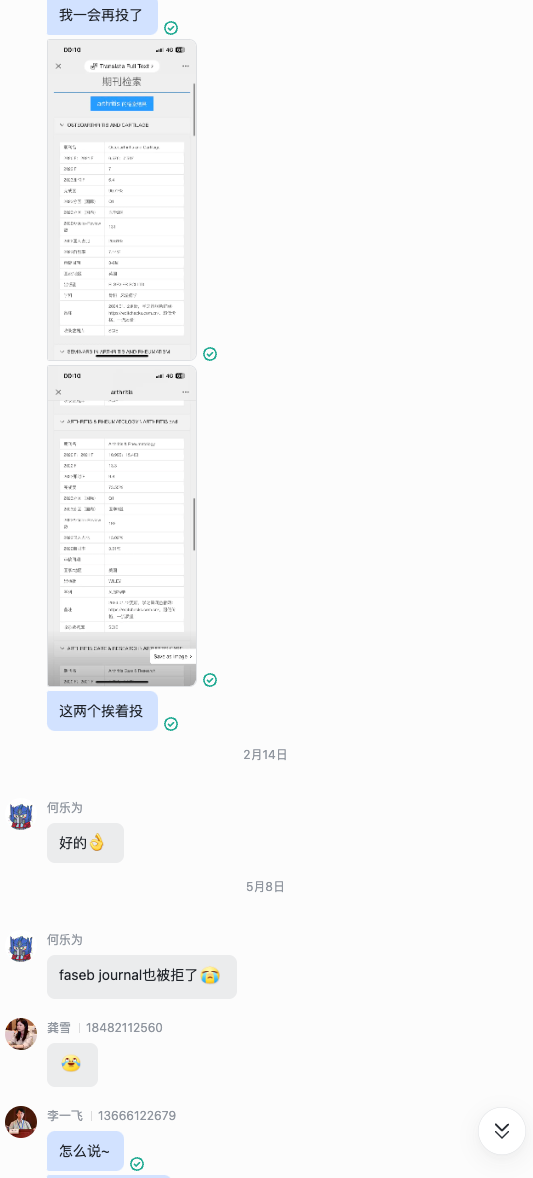


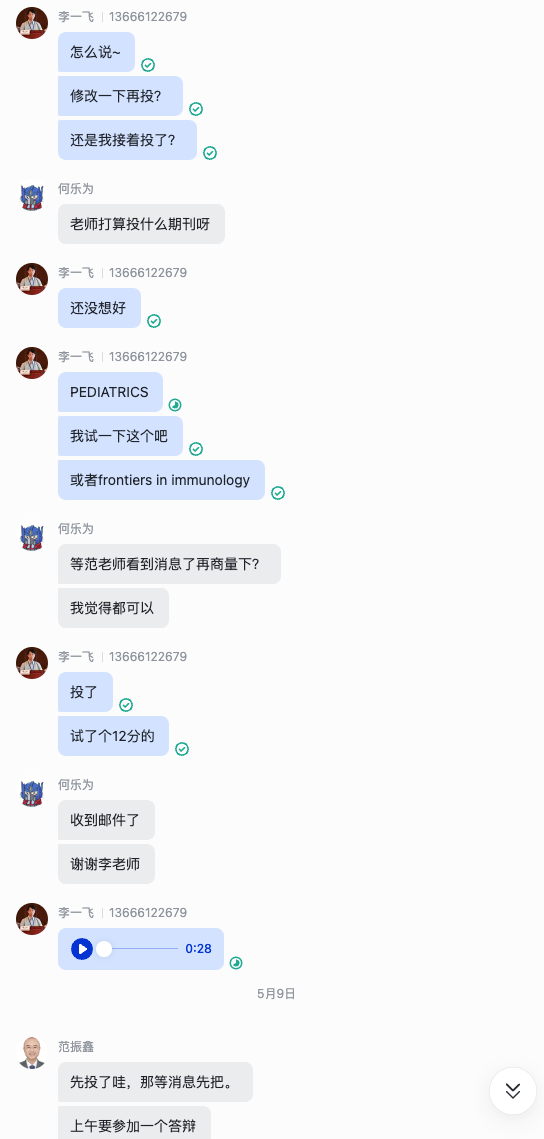


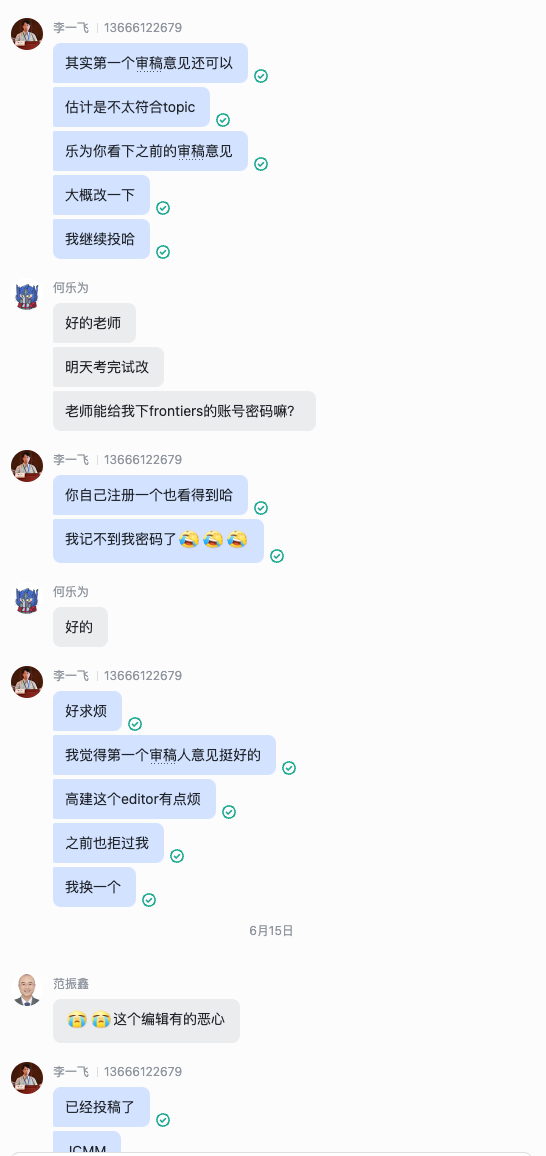

Supplement: Supplementary file 3 [file Table2.docx]
