# Supplementary material for: Single cell RNA-sequencing identified CCR7+/RELB+/IRF1+ T cell responding for juvenile idiopathic arthritis pathogenesis
Source: Front Immunol. 2025 May 8;16:1528446. doi: 10.3389/fimmu.2025.1528446 (PMC12095314; doi:10.3389/fimmu.2025.1528446)
Supplement: Supplementary file 6 [file Table5.docx]

**Single cell RNA-sequencing identified CCR7^+^/RELB^+^/IRF1^+^ T cell responding for juvenile idiopathic arthritis pathogenesis**

Running title: ScRNA-seq for JIA

Lewei He^1^#, Xue Gong^1^#, Hui Guo^1^#, Zhenxin Fan^1^#, Kaiyu Zhou^1^, Yue Lan^1^, Mingyi Lv^1^, Xiaoliang Liu^1^, Sha Lin^1^, Yimin Hua^1^, Junling Guo^2^, Yifei Li^1^†

1. Key Laboratory of Bioresources and Eco-Environment of MOE, College of Life Sciences, Key Laboratory of Birth Defects and Related Diseases of Women and Children of MOE, Department of Pediatrics, West China Second University Hospital, Sichuan University, Chengdu, Sichuan 610041, China.

2. BMI Center for Biomass Materials and Nanointerfaces, College of Biomass Science and Engineering, Sichuan University, Chengdu, Sichuan 610065, China.

# These authors contributed equally to this work.

**†Correspondence to:**

Yifei Li, MD, Key Laboratory of Birth Defects and Related Diseases of Women and Children of MOE, Department of Pediatrics, West China Second University Hospital, Sichuan University, 20 3rd Section, Renmin S.Rd., Chengdu, Sichuan, 610041. Tel: 86-181-8060-1016. Email: [liyfwcsh@scu.edu.cn](mailto:liyfwcsh@scu.edu.cn).

**Funding**: YL was funded by National Natural Science Foundation of China (grant no. 82270249).

**Competing interests**: The authors declare no competing interests.

**Keywords**: JIA; scRNA-seq; T cells; CCR7; autoimmune diseases.

**Abstract**

**Background:** Juvenile idiopathic arthritis (JIA) is a rare autoimmune related arthritis in childhood. The presence or absence of human leukocyte antigen (HLA-B27) in JIA suggests disease heterogeneity with unknown diverse immunopathological mechanisms in the disease. We use single-cell transcriptome sequencing to analyze the immune cellular features and pathogenesis mechanisms in JIA.

**Methods:** The single-cell RNA sequencing (scRNA-seq) analysis of peripheral blood mononuclear cells (PBMCs) was carried out to investigate the disease heterogeneity and molecular mechanisms of immune responses in immune cells in JIA.

**Results:** In our study, we provided an immunological landscape of HLA-B27-positive JIA and HLA-B27-negative JIA immune cells at a single cell RNA-Seq resolution. We found a higher proportion of CCR7+/RELB+/IRF1+ triple positive T cells in the peripheral blood of patients with JIA, and such T cells were predominantly present in HLA-B27^+^ JIA patients. Furthermore, we identified CCR7+/RELB+/IRF1+ triple positive T cells as highly activated T cells capable of promoting the differentiation of osteoclasts by producing IL-17. Therefore, CCR7+/RELB+/IRF1+ triple positive T cells, which were abundant in HLA-B27^+^ JIA patients, played a critical role in the pathogenesis of JIA. Our results also showed that different types of B cells (Naïve B cells and Memory B cells) and Monocytes (CD14+ Monocytes and CD16+ Monocytes) were involved in JIA.
**Conclusions:** Our scRNA-seq analysis clarified the heterogeneity of immune cells in patients with different subtypes of JIA and delineated the immune microenvironment in the peripheral blood of JIA patients, which may provide novel therapeutic targets of JIA.

**Introduction**

Juvenile idiopathic arthritis (JIA) is a kind of rare autoimmune disease which had been considered only involved in children. Although cartilage degradation has been demonstrated as the dominant pathophysiological process in JIA. Thus, it is critical to demonstrate the etiology of JIA. It was reported that immune cells in peripheral blood had a clear role in mediating joint injury, but the pathogenesis in JIA had not yet been well understood(1). Besides, some studies demonstrated abnormal immune cells participated in arthritis as a major factor (2, 3). Due to the great limitation in determining the specific cellular subtypes of immune cells, it is rarely possible to explore the potential the heterogeneities of immune cell in JIA children compared to normal ones, which limits the essential molecular mechanisms in regulating JIA immune responses to be addressed.

HLA-B27 is a human leukocyte antigen (HLA) protein encoded by the HLA-B gene. It's a crucial part of the immune system. HLA-B27 is found on the surface of most cells in the body and plays a significant role in presenting protein fragments to immune cells called T lymphocytes, determining different classification and treatment (4-7). However, it is still unknown that how HLA-B27 influences the immune cells developmental maturation and molecular function. Therefore, it is necessary to further elucidate the molecular mechanism of JIA pathogenesis under the influence of HLA-B27.

Currently, the single-cell RNA sequencing (scRNA-seq) made a great contribution in underline the heterogeneities among analyzed cells. Herein, we applied scRNA-Seq to explore peripheral blood mononuclear cells (PBMCs) composition, proportion, gene expression characteristics, and developmental trajectory in JIA cases and the potential roles of HLA-B27. Moreover, the scRNA-seq data of PBMCs of primary Sjögren's syndrome (pSS) and systemic lupus erythematosus (SLE) had been involved to validate the findings presented a high specificity in JIA, which was tried to identify the particular molecular function of JIA among other related autoimmune diseases.

**Methods**

**Ethical approval**

Patients with autoimmune disease before JIA onset, monoclonal antibody therapy, or other blood diseases were excluded. Participants’ parents or guardians provided informed consent to participate in this study. Patient data were collected by Yifei Li. And this study was approved by the Ethics Committee of West China Second Hospital of Sichuan University (2021-069).

**Sample preparation for 10x Genomics**

Seven children were diagnosed as JIA, three patients with HLA-B27- and four patients with HLA-B27+. Three age-matched volunteers were enrolled as children healthy control (cHC) were collected. The published data on scRNA-seq of PBMCs of 5 patients with pSS (pSS) (GSE157278) (8), 4 patients with SLE (SLE) (GSE137029) (9) and 5 adult healthy control ones (aHC) (GSE157278) (8) had been reanalyzed.

**Single-cell mRNA sequencing and data analysis**

Peripheral blood samples (4 mL each sample) were collected from the ten subjects. The single-cell suspensions of scRNA-seq samples were converted to barcoded scRNA-seq libraries using the Chromium Single Cell 5′ Library, Gel Bead and Multiplex Kit, and Chip Kit (10x Genomics). The Chromium Single Cell 5′ v2 Reagent (10x Genomics) kit was used to prepare single-cell RNA libraries according to the manufacturer's instructions. The data analysis involved quality control, cell type annotation, enrichment analysis, Cell trajectory analysis, Cell-cell interaction analysis and visualization(10-16). The detail information had been provided in Supplementary Materials.

**Results**

**The immunological landscape of JIA and cHC immune cells**

To investigate the differences of immune cells between JIA patients and healthy controls, we performed scRNA-seq on PBMCs from JIA patients (JIA, n=7) and age-matched healthy children controls (cHC, n=3) (Fig 1A). After standard data preprocessing, we obtained a single-cell transcriptome of a total of 97,208 immune cells from JIA and cHC (Fig 1B, C).

Six separated T cell subtypes, three B cell subtypes and four Myeloid cell subtypes had been identified from JIA and cHC (Fig 1D, E). A preliminary comparsion of the proportion of cells in JIA and cHC revealed significant heterogeneity in the proportion of T cell subtypes between the two groups. CD8 TEM and CCR7^+^ T cells compositions presented higher significant elevated ration among JIA cases compared to cHC ones (Fig 1F). However, there was no significant difference in the proportions of B cell subtypes and myeloid cell subtypes between JIA and cHC (Fig 1F).

Investigating molecular functional shifts within various immune cell types in JIA, we conducted statistical analyses on DEGs across diverse cellular categories between JIA and cHC (Fig 1G). Our findings revealed that among T cell subtypes, CCR7^+^ T cells exhibited the highest count of DEGs (Fig 1G). Gene Ontology (GO) enrichment analysis of these DEGs originating from distinct immune cell types demonstrated that upregulated DEGs in JIA significantly enriched GO terms associated with immune function. Conversely, downregulated DEGs in JIA displayed notable enrichment in GO terms linked to growth and development (Fig 1G). Further exploration aimed at pinpointing the most responsive immune cell type to JIA involved the application of the Augur algorithm to our scRNA-Seq data. The analysis suggested that JIA potentially exerts the most pronounced influence on CCR7^+^ T cells compared to cHC (AUC > 0.9) (Fig 1H).

**CCR7+/RELB+/IRF1+ T cells contributed in the JIA as a dominant cluster**

The UMAP projection vividly illustrated the distribution of T cells across JIA patients and cHC (Fig S1A). Within the JIA and cHC datasets, the T cells in both groups were categorized into six subtypes (Fig 2A). Our analysis delved into the developmental trajectories of T cells in JIA and cHC, unveiling distinct differences. Within JIA patients, the trajectory of T cell differentiation revealed an initial alteration in T cell homeostasis, followed by activation with enhanced expression of T cell receptors, culminating in the onset of a T cell-mediated inflammatory response (Fig 2B). Of significance during the differentiation of T cells in JIA, a notable emergence of CCR7^+^ T cells was observed at an early stage (Fig 2C, S2B).

To uncover the distinct mechanisms underlying the involvement of CCR7^+^ T cells in JIA, we separately analyzed their DEGs compared to CCR7^+^ T cells from cHC and CCR7^-^ T cells from JIA (Fig 2D). Notably, the upregulated DEGs in both comparisons significantly enriched pathways related to Osteoclast differentiation (KEGG:04380) and T cell activation (GO:0042110) (Fig 2E). Within these DEGs, *RELB* and *IRF1* emerged as pivotal genes involved in osteoclast differentiation and T cell activation, respectively. Volcano plots highlighted the substantial differential expression and significance of *RELB* and *IRF1* in JIA's CCR7^+^ T cells compared to cHC's T cells (Fig 2F). Violin plots depicted distinct expression levels of *RELB* and *IRF1* genes across different T cell subtypes (Fig 2G), revealing significantly elevated expression in JIA's CCR7^+^ T cells compared not only to cHC's CCR7^+^ T cells but also to other T cells in JIA (Fig 2G). Isolation of CCR7+/RELB+/IRF1+ triple positive T cells from JIA patients and cHC individuals confirmed a presence in JIA's CCR7^+^ T cells, whereas cHC exhibited this triple positivity in subsets of Naïve T cells (Fig 2H, S2A). Importantly, the proportion of T cells exhibiting CCR7+/RELB+/IRF1+ triple positivity was significantly higher in JIA compared to cHC (Fig S2A). Further investigation of gene expression and functionality of CCR7+/RELB+/IRF1+ T cells in JIA versus other CCR7^+^ T cells revealed significantly enriched upregulated DEGs associated with cytokine production (GO:0001816), TNF signaling pathway (KEGG:04668), and IL-17 signaling pathway (KEGG:04657) (Fig 2I). Specifically, elevated expression levels of cytokine-related genes *NFKB1* and *NFKB2* were observed in JIA's CCR7^+^/RELB^+^/IRF1^+^ T cells compared to other CCR7^+^ T cells from JIA (Fig S3).

**HLA-B27^+^ aggravated T cells mediating immune responses in JIA**

Though CCR7+/RELB+/IRF1+ T cells were observed notably in JIA patients, the molecular role of HLA-B27 in regulating T cell formation and its involvement in immune cell functionality maintenance remained unknown. Our JIA samples (n=7) comprised both HLA-B27^-^ (n=3) and HLA-B27^+^ (n=4) subtypes. Comparison of T cell subtype proportions between HLA-B27^+^ and HLA-B27^-^ JIA revealed a predominant presence of CCR7^+^ T cells in HLA-B27^+^ JIA, with minimal representation in HLA-B27^-^ JIA (Fig 3A, B, S5A). Further investigation into the differentiation trajectories of T cells between HLA-B27^-^ and HLA-B27^+^ JIA revealed a substantial appearance of CCR7^+^ T cells in the early stages of T cell differentiation (Fig 3C, S5B). In contrast, HLA-B27^-^ JIA exhibited a limited presence of CCR7^+^ T cells during T cell differentiation compared to HLA-B27^+^ JIA (Fig 3C, S5B). Notably, in HLA-B27^-^ JIA, T cells in both early and late stages of differentiation upregulated a greater number of genes associated with the immune response. Conversely, in HLA-B27^+^ JIA, T cells at the late stage of differentiation exhibited increased upregulation of immune response-related genes (Fig S5C).

To delve deeper into the gene expression variances among T cells in HLA-B27^+^ JIA and HLA-B27^-^ JIA, we examined the differential expression and functionality of upregulated DEGs in T cells from both HLA-B27^+^ JIA and HLA-B27^-^ JIA compared to T cells from cHC. Notably, the upregulated DEGs in both HLA-B27^+^ and HLA-B27^-^ JIA prominently enriched pathways associated with T cell receptor (TCR) signaling (GO:0050852) and cytokine production (GO:0001816) (Fig 3D). However, differences emerged; cell communication (GO:0007154) exhibited higher enrichment in HLA-B27^-^ JIA T cells, whereas NF-kappaB complex (GO:0071159) showed significant enrichment solely in HLA-B27^+^ JIA T cells (Fig 3D). Further exploration focused on the expression levels of TCR-associated genes in HLA-B27^+^ JIA and HLA-B27^-^ JIA T cells compared to cHC, revealing notably higher *EZR* expression in HLA-B27^+^ JIA T cells compared to HLA-B27^-^ JIA (Fig 3E).

Employing CellphoneDB, we investigated the interplay between T cells and other immune cell types in both HLA-B27^+^ JIA and HLA-B27^-^ JIA (Fig 3F). Heatmaps illustrating interaction intensity between different immune cell types revealed that CCR7^+^ T cells in both JIA subtypes, including CCR7+/RELB+/IRF1+ T cells, and CCR7^+^ T cells in cHC exhibited relatively weak interactions with other immune cell types (Fig 3F). Based on these interactions, we hypothesized that CCR7+/RELB+/IRF1+ T cells may not damage HLA-B27^+^ JIA cartilage through interactions with other immune cells.

To further discern the contrasting cytokine production capabilities of T cells in HLA-B27^+^ and HLA-B27^-^ JIA, we employed gene score profiles. Notably, T cells in HLA-B27^+^ JIA displayed elevated profile scores for IL-17 and TNF, while T cells in HLA-B27- JIA showcased higher profile scores for IL-2 and NF-kappaB (Fig 3G). We presented violin plots illustrating the expression patterns of 10 representative genes (Fig 3H, S5). Among these, *TNF*, *NFKB1*, and *NFKB2* exhibited predominant expression in T cells of HLA-B27^+^ JIA. Meanwhile, the expression of *IFNγ* did not significantly differ between T cells of HLA-B27^+^ JIA and HLA-B27^-^ JIA (Fig 3H, S5).

**CCR7+/RELB+/IRF1+ T cells independently response for JIA**

To discern differences in immune cell compositions across various autoimmune diseases, including JIA, pSS, and SLE, we opted to analyze PBMCs obtained from primary Sjögren's syndrome (pSS, n=5), systemic lupus erythematosus (SLE, n=4), and adult healthy controls (aHC, n=5), in conjunction with PBMCs from JIA and cHC. Following data preprocessing, we acquired a single-cell transcriptome of 137,454 immune cells (Fig S6A). Distinguishing from JIA and cHC, we identified three additional T cell subtypes—CD4 TEM (CD4^+^ Effector Memory T cells), MAIT (Mucosal-associated invariant T cells), and PTPRC^+^ T (PTPRC^+^ T cells)—in pSS, SLE, and aHC (Fig 4A, S6B). Comparing cell subtype proportions of T cells, B cells, and Myeloid cells among patients with different diseases and healthy controls revealed the exclusive presence of CCR7^+^ T cells in JIA patients and cHC (Fig 4A, S7A, B). Further analysis aimed to pinpoint the immune cell types most responsive to JIA, pSS, and SLE involved employing the Augur algorithm on scRNA-Seq data, focusing on the shared immune cells among JIA, pSS, and SLE (Fig 4B). Our findings indicated that pSS potentially exerts a significant impact on CD4 Naïve and CD8 Naïve cells compared to aHC (AUC > 0.8). Conversely, SLE demonstrated a notable impact on DC and CD16 Mono cells compared to aHC (AUC > 0.96) (Fig 4B).

We analyzed the upregulated DEGs specific to the T cell subtypes among patients with JIA, pSS and SLE, comparing JIA with cHC, pSS with aHC, and SLE with aHC, respectively (Fig 4C). Subsequently, we conducted GO terms and KEGG pathways enrichments based on the upregulated DEGs within different autoimmune diseases (Fig 4D). Remarkably, the TCR signaling pathway (GO:0050852) and cytokine production (GO:0001816) were significantly enriched among upregulated DEGs in JIA, pSS, and SLE T cells (Fig 4D). Moreover, the expression level of *EZR* in JIA T cells significantly surpassed that in pSS and SLE (Fig 4E). Exploration of *RELB* and *IRF1* expression on T cells from JIA, pSS, and SLE revealed lower levels in pSS and SLE T cells compared to aHC (Fig S7C). Across the T cell populations of these autoimmune diseases, *CCR7*, *RELB*, and *IRF1* were notably highly expressed in JIA T cells (Fig 4F), which revealed that CCR7+/RELB+/IRF1+ T cells independently response for JIA. Additionally, a preliminary analysis of immune cell interactions among patients with these autoimmune diseases revealed minimal interaction between T cell subtypes and other immune cell types (Fig 4G).

We employed gene score profiles to delve deeper into the differences in cytokine production abilities of T cells in JIA compared to those in pSS and SLE. The results revealed that both T cells and CD4 T cells in JIA exhibited a potentially heightened capacity to produce IL-17, while T cells, especially CD4 T cells, in pSS displayed increased potential for IL-2, TNF, and NF-kappaB production (Fig 4H, S7B, S7C). Subsequently, we calculated shared and non-shared upregulated DEGs in T cells and CD4 T cells among JIA, pSS, and SLE (Fig S7A). Their capacity to produce IFN-β, IFN-γ, and NF-kappaB was compared by examining the upregulated DEGs in T cells across the three autoimmune diseases. Notably, T cells in JIA, pSS, and SLE exhibited robust capability in producing NF-kappaB, IFN-γ, and IFN-β (Fig 4I).

**Characteristics of B cells between HLA-B27^-^ and HLA-B27^+^ JIA**

The UMAP projection illustrated the distribution of B cell subtypes among various JIA patients, including HLA-B27^+^ JIA, HLA-B27^-^ JIA, and cHC (Fig 5A). Within JIA and cHC, B cells were categorized into three subtypes (Fig S9A). The differentiation trajectories of B cells from JIA exhibited no anomalies (Fig S9B). Further subdivision of B cells in HLA-B27^+^ JIA and HLA-B27^-^ JIA also revealed three subtypes (Fig S9C). Notably, Plasma B in HLA-B27^-^ JIA emerged early in development, whereas in HLA-B27^+^ JIA, they appeared late in development (Fig 5B, S9D). At the early stage of differentiation, Plasma B in HLA-B27^-^ JIA showed increased upregulation of genes related to the immune response. In contrast, B cells in HLA-B27^+^ JIA, during both early and late differentiation stages, displayed elevated expression of genes associated with the immune response (Fig 5C).

We conducted a comprehensive analysis of upregulated DEGs in B cell subtypes from both HLA-B27^-^ JIA and HLA-B27^+^ JIA compared to cHC (Fig 5D). These DEGs showed significant enrichment in immune-related GO terms, including immune system processes (GO:0002376), immune response (GO:0006955), and B cell activation (GO:0042113) (Fig 5E). Notably, DEGs exclusively upregulated in Naïve B of both HLA-B27^-^ JIA (n=178, 49.3%) and HLA-B27^+^ JIA (n=104, 28.8%), as well as those co-upregulated by Naïve B in both conditions (n=79, 21.9%), exhibited significant enrichment in MHC class II protein complex (GO:0042613) (Fig 5D, E). Furthermore, DEGs co-upregulated by Memory B in HLA-B27^-^ JIA and HLA-B27^+^ JIA (n=271, 47.7%) were notably enriched in the B cell receptor signaling pathway (GO:0050853) (Fig 5D, E). We visualized the gene expression of the B cell receptor signaling pathway using a heatmap, revealing slightly more B cell receptor signaling-associated DEGs in Memory B from HLA-B27^-^ JIA than in those from HLA-B27^+^ JIA (Fig 5F). Noteworthy differences emerged in the expression levels of specific genes: Naïve B of HLA-B27^-^ JIA showed elevated levels of *HLA-DMB*, *HLA-DOB*, and *B2M*, whereas Naïve B of HLA-B27^+^ JIA displayed increased expression of *HLA-DPB1*, *HLA-DQA2*, and *HLA-DQB1* (Fig 5G). Additionally, both Naïve B populations in HLA-B27^-^ JIA and HLA-B27^+^ JIA exhibited significantly heightened expression levels of *HLA-DRA*, *HLA-DRB1*, and *CD74* (Fig 5G).

**Characteristics of Myeloid cells between HLA-B27^-^ and HLA-B27^+^ JIA**

The UMAP projection illustrated the distribution of Myeloid cell subtypes among JIA patients, encompassing HLA-B27^+^ JIA, HLA-B27^-^ JIA, and cHC (Fig 6A). An analysis comparing the differentiation trajectories of monocytes in JIA versus cHC revealed minimal divergence between them (Fig S10A). Further exploration of the differentiation trajectories of monocytes between HLA-B27^-^ JIA and HLA-B27^+^ JIA highlighted distinct patterns (Fig 6B, S10B). Notably, CD16 Mono in HLA-B27^-^ JIA emerged early in differentiation, while Inter Mono appeared later in HLA-B27^-^ JIA, contrasting with early emergence in HLA-B27^+^ JIA (Fig 6B, S10B). Interestingly, CD14 Mono and CD16 Mono in HLA-B27^-^ JIA exhibited increased upregulation of genes related to the immune response at the early stage of differentiation, whereas in HLA-B27^+^ JIA, these cell types showed greater upregulation of immune response-related genes at the middle stage of differentiation (Fig S10C).

The UMAP projection illustrated the distribution of Myeloid cell subtypes among JIA patients, encompassing HLA-B27^+^ JIA, HLA-B27^-^ JIA, and cHC (Fig 6A). An analysis comparing the differentiation trajectories of monocytes in JIA versus cHC revealed minimal divergence between them (Fig S10A). Further exploration of the differentiation trajectories of monocytes between HLA-B27^-^ JIA and HLA-B27^+^ JIA highlighted distinct patterns (Fig 6B, S10B). Notably, CD16 Mono in HLA-B27^-^ JIA emerged early in differentiation, while Inter Mono appeared later in HLA-B27^-^ JIA, contrasting with early emergence in HLA-B27^+^ JIA (Fig 6B, S10B). Interestingly, CD14 Mono and CD16 Mono in HLA-B27^-^ JIA exhibited increased upregulation of genes related to the immune response at the early stage of differentiation, whereas in HLA-B27^+^ JIA, these cell types showed greater upregulation of immune response-related genes at the middle stage of differentiation (Fig S10C).

We conducted an analysis of upregulated DEGs in all Myeloid cell subtypes of HLA-B27^-^ JIA and HLA-B27^+^ JIA compared to cHC (Fig 6C). Specifically, the upregulated DEGs in CD14 Mono and CD16 Mono from both HLA-B27^-^ JIA and HLA-B27^+^ JIA demonstrated significant enrichment in immune-related GO terms, including immune system processes (GO:0002376), immune response (GO:0006955), and MAPK cascade (GO:0007255) (Fig 6D). Further GO enrichment analysis revealed that DEGs co-upregulated by CD14 Mono in both HLA-B27^-^ JIA and HLA-B27^+^ JIA (n=133, 30.2%) were notably enriched in IL-1 beta production (GO:0050720), IL-6 production (GO:0042226), and IL-8 production (GO:0042228). Intriguingly, DEGs exclusively upregulated in CD14 Mono of HLA-B27- JIA (n=190, 43.2%) also displayed significant enrichment in IL-6 production (GO:0042226) and IL-8 production (GO:0042228) (Fig 6E, F). Subsequent comparison of the IL production capacity of CD14 Mono in HLA-B27^-^ JIA and HLA-B27^+^ JIA through heatmap and Sankey diagram analysis revealed that CD14 Mono in both conditions produced IL-1β, IL-6, and IL-8. Notably, HLA-B27- JIA demonstrated a higher number of upregulated DEGs associated with IL-6 production (GO:0042226) and IL-8 production (GO:0042228), indicating a potentially heightened capacity for pro-inflammatory interleukin production in CD14 Mono from HLA-B27^-^ JIA (Fig 6E, F).

We delved into the DEGs and GO enrichment results focused on CD16 Mono in both HLA-B27^-^ JIA and HLA-B27^+^ JIA. Notably, the DEGs co-upregulated by CD16 Mono in both conditions (n=324, 45.7%) displayed significant enrichment in IL-1 beta production (GO:0050720), IL-6 production (GO:0042226), and IL-8 production (GO:0042228). Additionally, DEGs exclusively upregulated in CD16 Mono of HLA-B27^-^ JIA (n=295, 41.6%) showed notable enrichment in IL-8 production (GO:0042228) (Fig 6C, D). Further comparison of the interleukin production capacity of CD16 Mono between HLA-B27^-^ JIA and HLA-B27^+^ JIA through heatmap and Sankey diagram analysis revealed that both subsets produced IL-1β, IL-6, and IL-8. Intriguingly, HLA-B27^-^ JIA displayed a higher count of upregulated DEGs associated with IL-8 production (GO:0042228), suggesting a potentially heightened ability for pro-inflammatory IL production in CD16 Mono from HLA-B27^-^ JIA (Fig 6E, F).

**Discussion**

JIA is a kind of autoimmune condition emerging before 16 years of age (1, 17). And HLA-B27, had been considered to be participated in the prognosis of JIA (18, 19). In JIA, HLA-B27 is acknowledged as a risk factor implicated in exacerbating arthritis severity(20). However, not all individuals with HLA-B27 develop JIA, and the delineating JIA into distinct clinical subtypes solely based on HLA-B27 status remains uncertain (21, 22). The bone loss observed in JIA often results in decreased bone mineral density (BMD) (23, 24), offering insights into JIA's etiology and pathogenesis. Dysregulated immune cells contribute to chronic inflammation and tissue damage, particularly in the joints, leading to the characteristic arthritis seen in JIA patients. Our study delved into JIA's pathogenesis, revealing a pivotal link between differential gene expression in T cells and bone damage, particularly in HLA-B27^+^ JIA.

This observation led us to hypothesize the pivotal role of CCR7^+^ T cells in JIA's pathogenesis. To validate this hypothesis, we examined the DEGs within CCR7^+^ T cells from JIA versus CCR7^+^ T cells from cHC and other T cells within JIA. The upregulated DEGs within CCR7^+^ T cells from JIA were notably associated with T cell activation and osteoclast differentiation (25, 26). T cells are instrumental in initiating immune responses, and their overactivation frequently underlies autoimmune diseases (27, 28). Moreover, cytokines derived from activated T cells have been linked to promoting osteoclast differentiation (29, 30). We delved deeper into the significantly upregulated DEGs within CCR7^+^ T cells from JIA. *RELB* has a known role in osteoclast generation, while IRF1's involvement in abnormal T cell activation contributes to various autoimmune conditions (31-34). Analyzing the upregulated DEGs by CCR7+/RELB+/IRF1+ T cells in JIA compared to other CCR7^+^ T cells revealed enrichment in the IL-17 and TNF signaling pathways. Both IL-17 and TNF are pivotal in autoimmune diseases like JIA and ankylosing spondylitis (35, 36). IL-17's direct promotion of osteoclast formation has been evident in rheumatoid arthritis models (37, 38). Consequently, our findings suggest a potential mechanism wherein CCR7+/RELB+/IRF1+ T cells might induce osteoclast differentiation via cytokine production, particularly IL-17, contributing to bone damage in JIA patients. Further exploration is warranted to fully elucidate this pathway's intricacies and potential therapeutic targets.

Following the categorization of JIA into HLA-B27^+^ and HLA-B27^-^ subsets, a notable prevalence of CCR7^+^ T cells was observed in JIA. HLA-B27 is closely linked to JIA, where its presence is associated with clinical sacroiliitis symptoms and impedes symptom remission (7, 39, 40). Among the genes involved in the TCR signaling pathway, increased expression of *EZR* was noticed in T cells of HLA-B27^+^ JIA. This encoded ezrin protein is known to interact with CD44, a significant contributor to osteoarthritis progression (41, 42). When comparing cytokine production between T cells in HLA-B27^+^ JIA and HLA-B27^-^ JIA, a higher capacity for IL-17 and TNF production was evident in T cells from HLA-B27^+^ JIA. Based on these findings, the upregulation of EZR expression by CCR7+/RELB+/IRF1+ T cells was observed. This potentially augments the interaction between CD44 and chondrocytes, known to produce substantial IL-17 amounts. Consequently, this cascade might trigger chondrocyte damage in JIA, especially within the context of HLA-B27^+^ JIA.

Then, we illustrated the characterization and gene expression of T cells in pSS and SLE, comparing them with those in JIA. Notably, CCR7^+^ T cells were absent in both pSS and SLE. Specific genes—*CCR7*, *RELB*, and *IRF1*—displayed heightened expression exclusively in T cells from JIA. Moreover, *EZR* expression levels were notably higher in T cells from JIA compared to those in pSS and SLE. T cells within JIA, including CD4 T cells, exhibited a greater capacity for IL-17 production than their counterparts in pSS and SLE. From these observations, we conjectured that the gene expression and functionality of CCR7+/RELB+/IRF1+ T cells could represent a distinctive feature of JIA, setting it apart from pSS and SLE and potentially serving as a therapeutic target for JIA.

**Conclusion**

In summary, this study provided a transcriptional landscape of immune cells in JIA patients at single cell resolution. The results initially identified that cluster of CCR7^+^/RELB^+^/IRF1^+^ T cells contributed dominantly in mediating the pathogenesis of JIA, leading to bone degradation by osteoclast overactivation. Moreover, the HLA-B27 aggregated the immune activity of CCR7^+^/RELB^+^/IRF1^+^ T cells, resulting in adverse prognosis for JIA individuals with HLA-B27^+^. Also, CCR7^+^/RELB^+^/IRF1^+^ T cells had been revealed as an independent responsor in JIA among other types of autoimmune diseases, severing as potential therapeutic target.

Availability of data and materials

The datasets analyzed during the current study are available in GSA (https://ngdc.cncb.ac.cn/gsub/, HRA006261).

**Competing interests**

The authors declare no competing interests.

**Funding**

Yifei Li was funded by National Natural Science Foundation of China (grant no. 82270249).

**Authors' contributions**

Yifei Li, ZF and JG conceptualized the study; LH, XG, HG, KZ, Yue Lan, ML, JG, ZF and Yifei Li designed the analysis; HG, XL, YH and Yifei Li were the physicians of the involved patients; LH, XG, Yue Lan, ML and ZF analyzed data; LH, XG, JG, ZF and Yifei Li wrote or edited the manuscript.

**Acknowledgements**

We acknowledge all patients and their families for the support of our study. We thank the support from the College of Life Sciences, Sichuan University and West China Second University Hospital.

**References**

1. Prakken B, Albani S, Martini A. Juvenile idiopathic arthritis. Lancet (London, England). 2011;377(9783):2138-49.

2. Bessis N, Decker P, Assier E, Semerano L, Boissier MC. Arthritis models: usefulness and interpretation. Semin Immunopathol. 2017;39(4):469-86.

3. Woodell-May JE, Sommerfeld SD. Role of Inflammation and the Immune System in the Progression of Osteoarthritis. J Orthop Res. 2020;38(2):253-7.

4. Adrovic A, Barut K, Sahin S, Kasapcopur O. Juvenile Spondyloarthropathies. Curr Rheumatol Rep. 2016;18(8):55.

5. Flatø B, Hoffmann-Vold AM, Reiff A, Førre Ø, Lien G, Vinje O. Long-term outcome and prognostic factors in enthesitis-related arthritis: a case-control study. Arthritis Rheum. 2006;54(11):3573-82.

6. Bryan AR, Rabinovich CE. Enthesitis-related arthritis: time to re-define? Curr Rheumatol Rep. 2014;16(12):466.

7. Berntson L, Nordal E, Aalto K, Peltoniemi S, Herlin T, Zak M, et al. HLA-B27 predicts a more chronic disease course in an 8-year followup cohort of patients with juvenile idiopathic arthritis. The Journal of rheumatology. 2013;40(5):725-31.

8. Hong X, Meng S, Tang D, Wang T, Ding L, Yu H, et al. Single-Cell RNA Sequencing Reveals the Expansion of Cytotoxic CD4(+) T Lymphocytes and a Landscape of Immune Cells in Primary Sjögren's Syndrome. Frontiers in immunology. 2020;11:594658.

9. Mandric I, Schwarz T, Majumdar A, Hou K, Briscoe L, Perez R, et al. Optimized design of single-cell RNA sequencing experiments for cell-type-specific eQTL analysis. Nature communications. 2020;11(1):5504.

10. Pan L, Dinh HQ, Pawitan Y, Vu TN. Isoform-level quantification for single-cell RNA sequencing. Bioinformatics (Oxford, England). 2022;38(5):1287-94.

11. Butler A, Hoffman P, Smibert P, Papalexi E, Satija R. Integrating single-cell transcriptomic data across different conditions, technologies, and species. Nature biotechnology. 2018;36(5):411-20.

12. McGinnis CS, Murrow LM, Gartner ZJ. DoubletFinder: Doublet Detection in Single-Cell RNA Sequencing Data Using Artificial Nearest Neighbors. Cell systems. 2019;8(4):329-37.e4.

13. Skinnider MA, Squair JW, Kathe C, Anderson MA, Gautier M, Matson KJE, et al. Cell type prioritization in single-cell data. Nature biotechnology. 2021;39(1):30-4.

14. Chicco D, Jurman G. A brief survey of tools for genomic regions enrichment analysis. Frontiers in bioinformatics. 2022;2:968327.

15. Qiu X, Mao Q, Tang Y, Wang L, Chawla R, Pliner HA, et al. Reversed graph embedding resolves complex single-cell trajectories. Nature methods. 2017;14(10):979-82.

16. Efremova M, Vento-Tormo M, Teichmann SA, Vento-Tormo R. CellPhoneDB: inferring cell-cell communication from combined expression of multi-subunit ligand-receptor complexes. Nature protocols. 2020;15(4):1484-506.

17. Zaripova LN, Midgley A, Christmas SE, Beresford MW, Baildam EM, Oldershaw RA. Juvenile idiopathic arthritis: from aetiopathogenesis to therapeutic approaches. Pediatric rheumatology online journal. 2021;19(1):135.

18. Chen B, Li J, He C, Li D, Tong W, Zou Y, et al. Role of HLA-B27 in the pathogenesis of ankylosing spondylitis (Review). Molecular medicine reports. 2017;15(4):1943-51.

19. Sorrentino R, Böckmann RA, Fiorillo MT. HLA-B27 and antigen presentation: at the crossroads between immune defense and autoimmunity. Molecular immunology. 2014;57(1):22-7.

20. Żuber Z, Turowska-Heydel D, Sobczyk M, Chudek J. Prevalence of HLA-B27 antigen in patients with juvenile idiopathic arthritis. Reumatologia. 2015;53(3):125-30.

21. Schiellerup P, Krogfelt KA, Locht H. A comparison of self-reported joint symptoms following infection with different enteric pathogens: effect of HLA-B27. The Journal of rheumatology. 2008;35(3):480-7.

22. Classification Criteria for Spondyloarthritis/HLA-B27-Associated Anterior Uveitis. American journal of ophthalmology. 2021;228:117-25.

23. Martini A, Lovell DJ, Albani S, Brunner HI, Hyrich KL, Thompson SD, et al. Juvenile idiopathic arthritis. Nature reviews Disease primers. 2022;8(1):5.

24. Brabnikova Maresova K, Jarosova K, Pavelka K, Stepan JJ. Bone status in adults with early-onset juvenile idiopathic arthritis following 1-year anti-TNFα therapy and discontinuation of glucocorticoids. Rheumatology international. 2013;33(8):2001-7.

25. Gorentla BK, Zhong XP. T cell Receptor Signal Transduction in T lymphocytes. Journal of clinical & cellular immunology. 2012;2012(Suppl 12):5.

26. Shah K, Al-Haidari A, Sun J, Kazi JU. T cell receptor (TCR) signaling in health and disease. Signal transduction and targeted therapy. 2021;6(1):412.

27. Rosetti F, Madera-Salcedo IK, Rodríguez-Rodríguez N, Crispín JC. Regulation of activated T cell survival in rheumatic autoimmune diseases. Nature reviews Rheumatology. 2022;18(4):232-44.

28. Sun Y, Zhu X, Chen X, Liu H, Xu Y, Chu Y, et al. The mediator subunit Med23 contributes to controlling T-cell activation and prevents autoimmunity. Nature communications. 2014;5:5225.

29. Karieb S, Fox SW. Suppression of T cell-induced osteoclast formation. Biochemical and biophysical research communications. 2013;436(4):619-24.

30. Fischer V, Haffner-Luntzer M. Interaction between bone and immune cells: Implications for postmenopausal osteoporosis. Seminars in cell & developmental biology. 2022;123:14-21.

31. Zhao Z, Hou X, Yin X, Li Y, Duan R, Boyce BF, et al. TNF Induction of NF-κB RelB Enhances RANKL-Induced Osteoclastogenesis by Promoting Inflammatory Macrophage Differentiation but also Limits It through Suppression of NFATc1 Expression. PloS one. 2015;10(8):e0135728.

32. Vaira S, Johnson T, Hirbe AC, Alhawagri M, Anwisye I, Sammut B, et al. RelB is the NF-kappaB subunit downstream of NIK responsible for osteoclast differentiation. Proceedings of the National Academy of Sciences of the United States of America. 2008;105(10):3897-902.

33. Chen J, Peng L, Zhao Z, Yang Q, Yin F, Liu M, et al. HDAC1 potentiates CD4 + T cell activation by inhibiting miR-124 and promoting IRF1 in systemic lupus erythematosus. Cellular immunology. 2021;362:104284.

34. Zhang Z, Shi L, Song L, Ephrem E, Petri M, Sullivan KE. Interferon regulatory factor 1 marks activated genes and can induce target gene expression in systemic lupus erythematosus. Arthritis & rheumatology (Hoboken, NJ). 2015;67(3):785-96.

35. Kostik MM, Makhova MA, Maletin AS, Magomedova SM, Sorokina LS, Tsukasaki M, et al. Cytokine profile in patients with chronic non-bacterial osteomyelitis, juvenile idiopathic arthritis, and insulin-dependent diabetes mellitus. Cytokine. 2021;143:155521.

36. Ahluwalia B, Moraes L, Magnusson MK, Öhman L. Immunopathogenesis of inflammatory bowel disease and mechanisms of biological therapies. Scandinavian journal of gastroenterology. 2018;53(4):379-89.

37. Kotake S, Udagawa N, Takahashi N, Matsuzaki K, Itoh K, Ishiyama S, et al. IL-17 in synovial fluids from patients with rheumatoid arthritis is a potent stimulator of osteoclastogenesis. The Journal of clinical investigation. 1999;103(9):1345-52.

38. Gravallese EM, Schett G. Effects of the IL-23-IL-17 pathway on bone in spondyloarthritis. Nature reviews Rheumatology. 2018;14(11):631-40.

39. Hersh AO, Prahalad S. Immunogenetics of juvenile idiopathic arthritis: A comprehensive review. Journal of autoimmunity. 2015;64:113-24.

40. Colbert RA. Classification of juvenile spondyloarthritis: Enthesitis-related arthritis and beyond. Nature reviews Rheumatology. 2010;6(8):477-85.

41. Roumier A, Olivo-Marin JC, Arpin M, Michel F, Martin M, Mangeat P, et al. The membrane-microfilament linker ezrin is involved in the formation of the immunological synapse and in T cell activation. Immunity. 2001;15(5):715-28.

42. Bai RJ, Liu D, Li YS, Tian J, Yu DJ, Li HZ, et al. OPN inhibits autophagy through CD44, integrin and the MAPK pathway in osteoarthritic chondrocytes. Frontiers in endocrinology. 2022;13:919366.

**Figure legends**

**Figure 1. Immune cells composition differs in peripheral blood from JIA patients and cHC.** **A.** Overview of the research workflow. cHC: children healthy controls; HLA-B27: Human leukocyte antigen B27; PBMCs: Peripheral blood mononuclear cells. **B.** UMAP projection of the JIA and cHC. **C.** Integrated UMAP graph of B cells, T cells, Myeloid cells, NK, ILC, Neutrophil and Platelet derived from our research, colored by cell types. Among the identified immune cells, 39, 615 cells from JIA and 17, 127 cells from cHC. **D.** Integrated UMAP graph of T cell subtypes, B cell subtypes and Myeloid cell subtypes, colored by cell subtypes. **E.** Dot plot showing the expression level of classical cell markers used to assign cell identity. **F.** The proportion of different subtypes of T cells, B cells and Myeloid cells. G. Upregulated and downregulated differentially expressed genes (DEGs) in immune cell subtypes of JIA compared to cHC and the corresponding GO enrichment results. **H.** The AUC score of Augur algorithm was used to rank the cell types.

**Figure 2. The proportion of CCR7+/RELB+/IRF1+ triple positive T cells is an important feature of JIA patients.** **A.** Integrated UMAP graph of T cell subtypes in JIA patients and cHC respectively, colored by T cell subtypes. **B.** Heatmap showing dynamic changes in gene expression in T cell subtypes of JIA patients. **C.** Differentiation trajectories of T cell subtypes in JIA patients and cHC, respectively. **D.** Venn plot for the overlap of the upregulated DEGs by CCR7+ T cells in JIA patients compared with CCR7+ T cells in cHC and other T cells in JIA patients, respectively. **E.** The GO enrichment results of the upregulated DEGs by CCR7+ T cells in JIA patients compared with CCR7+ T cells in cHC and other T cells in JIA patients, respectively. **F.** Volcano plot showing differentially expressed genes (DEGs) between CCR7+ T cells in JIA and CCR7+ T cells in cHC. **G.** Violin plots showing the differences in expression levels of RELB and IRF1 between CCR7+ T cells in cHC and CCR7+ T cells in JIA patients; And the differences in expression levels of RELB and IRF1 between CCR7+ T cells in JIA patients and other T cells in JIA patients. **H.** Dispersed expression of CCR7, RELB and IRF1 cell populations. **I.** The GO and KEGG enrichment results of the upregulated DEGs by CCR7+/RELB+/IRF1+ triple positive T cells in JIA patients compared with other CCR7+ T cells in JIA patients. P values were calculated by Wilcox test. *p < 0.05, **p < 0.01, ***p < 0.001.

**Figure 3. CCR7+/RELB+/IRF1+ triple positive T cells are mainly present in T cells from HLA-B27+ JIA patients.** **A.** Integrated UMAP graph of T cell subtypes in HLA-B27+ JIA patients and HLA-B27- JIA patients, colored by T cell subtypes. **B.** The proportion of different subtypes of T cells between HLA-B27+ JIA patients and HLA-B27- JIA patients. **C.** Differentiation trajectories of T cell subtypes in HLA-B27+ JIA patients and HLA-B27- JIA patients, respectively. **D.** The GO enrichment results of the upregulated DEGs by T cells in HLA-B27- JIA and HLA-B27+ JIA compared with cHC, respectively. **E.** Heatmap of the gene expression of TCR signaling pathway in T cells between HLA-B27- JIA patients and HLA-B27+ JIA patients. **F.** Heatmaps showing the interaction between immune cells in HLA-B27+ JIA, HLA-B27- JIA and cHC, respectively. **G.** Profile scores of IL-2, IL-17, TNF and NF-kappaB in T cells among three groups. **H.** Violin plots showing the differences in expression levels of IL2, TNF, IFNγ, NFKB1 and NFKB2 in T cells from HLA-B27+ JIA, HLA-B27- JIA and cHC. P values were calculated by Wilcox test. *p < 0.05, **p < 0.01, ***p < 0.001.

**Figure 4. pSS patients and SLE patients lack T cells that express high levels of RELB and IRF1.** **A.** Integrated UMAP graph of 13,7454 immune cells derived from our study, colored by cell types. Among the identified immune cells, 24,962 cells from pSS, 32,655 cells from SLE and 23,095 cells from aHC. Bar plot showing the proportion of different subtypes of T cells, B cells and Myeloid cells. **B.** Augur algorithm was used to rank the cell types of JIA patients, pSS patients and SLE patients. **C.** Upregulated DEGs in T cell subtypes of JIA patients compared with cHC, and upregulated DEGs in T cell subtypes of pSS patients and SLE patients compared with aHC. **D.** GO enrichment results of upregulated DEGs in T cell subtypes of JIA patients, pSS patients and SLE patients. **E.** Heatmap of the gene expression of TCR signaling pathway of T cells in JIA patients, pSS patients and SLE patients. **F.** Polar plot gene expression across JIA, pSS and SLE for T cells. Off-axis points reflect shared expressions among patients with different autoimmune diseases. **G.** Heatmaps showing the interaction between immune cells in HLA-B27+ JIA, HLA-B27- JIA and cHC, respectively. **H.** Profile scores of IL-2, IL-17, TNF and NF-kappaB in T cells and CD4 T cells among three groups. **I.** Sankey diagrams showing the difference in IFN-β, IFN-γ and NF-kappaB of T cells in JIA patients, pSS patients and SLE patients. P values were calculated by Wilcox test. *p < 0.05, **p < 0.01, ***p < 0.001.

**Figure 5. Different B cell subtypes participate in JIA.** **A.** UMAP projections of B cells from JIA patients and cHC. **B.** Differentiation trajectories of B cell subtypes in JIA patients and cHC, respectively. **C.** Heatmaps showing dynamic changes in gene expression in B cell subtypes of HLA-B27+ JIA patients and HLA-B27- JIA patients, respectively. **D.** Venn plots for the overlaps of the upregulated DEGs between HLA-B27- JIA patients and HLA-B27+ JIA patients in Naive B and Memory B. **E.** The GO enrichment results of the upregulated genes between HLA-B27- JIA patients and HLA-B27+ JIA patients in Naive B and Memory B. **F.** Heatmap of the gene expression of BCR signaling pathway in Memory B between HLA-B27- JIA patients and HLA-B27+ JIA patients. **G.** Violin plots showing the differences in gene expression of MHC class II protein complex in PBMC for Naive B of HLA-B27- JIA patients and HLA-B27+ JIA patients. P values were calculated by Wilcox test. *p < 0.05, **p < 0.01, ***p < 0.001.

**Figure 6. Different Myeloid cell subtypes participate in JIA.** **A.** UMAP projections of Myeloid cells from JIA patients and cHC. **B.** Differentiation trajectories of Monocyte subtypes in HLA-B27+ JIA patients and HLA-B27- JIA patients, respectively. **C.** Venn plots for the overlaps of the upregulated DEGs between HLA-B27- JIA patients and HLA-B27+ JIA patients in CD14 Mono and CD16 Mono. **D.** The GO enrichment results of the upregulated DEGs between HLA-B27- JIA patients and HLA-B27+ JIA patients in CD14 Mono and CD16 Mono. **E.** Heatmaps of the gene expression of Interleukin production in CD14 Mono and CD16 Mono between HLA-B27- JIA patients and HLA-B27+ JIA patients. **F.** Sankey diagrams showing the difference in the pro-inflammatory interleukin between CD14 Mono and CD16 Mono for HLA-B27- JIA patients and HLA-B27+ JIA patients.
