# Supplementary material for: Single cell RNA-sequencing identified CCR7+/RELB+/IRF1+ T cell responding for juvenile idiopathic arthritis pathogenesis
Source: Front Immunol. 2025 May 8;16:1528446. doi: 10.3389/fimmu.2025.1528446 (PMC12095314; doi:10.3389/fimmu.2025.1528446)
Supplement: Supplementary file 7 [file Table6.docx]

**Single cell RNA-sequencing identified CCR7^+^/RELB^+^/IRF1^+^ T cell responding for juvenile idiopathic arthritis pathogenesis**

Lewei He^1^#, Xue Gong^1^#, Hui Guo^1^#, Kaiyu Zhou^1^, Yue Lan^1^, Mingyi Lv^1^, Yimin Hua^1^, Junling Guo^2^, Zhenxin Fan^1^†, Yifei Li^1^†

1. Key Laboratory of Bioresources and Eco-Environment of MOE, College of Life Sciences, Key Laboratory of Birth Defects and Related Diseases of Women and Children of MOE, Department of Pediatrics, West China Second University Hospital, Sichuan University, Chengdu, Sichuan 610041, China.

2. BMI Center for Biomass Materials and Nanointerfaces, College of Biomass Science and Engineering, Sichuan University, Chengdu, Sichuan 610065, China.

# These authors contributed equally to this work.

**†Correspondence to:**

Zhenxin Fan, PhD, Key Laboratory of Bioresources and Eco-Environment of MOE, College of Life Sciences, Sichuan University, Chengdu, China. Tel: 86-136-6616-7984. Email: zxfan@scu.edu.cn;

Yifei Li, MD, Key Laboratory of Birth Defects and Related Diseases of Women and Children of MOE, Department of Pediatrics, West China Second University Hospital, Sichuan University, 20 3rd Section, Renmin S.Rd., Chengdu, Sichuan, 610041. Tel: 86-181-8060-1016. Email: [liyfwcsh@scu.edu.cn](mailto:liyfwcsh@scu.edu.cn). (Leading contact)

Conflict-of-interest statement: The authors have declared that no conflict of interest exists.

**Abstract**

Juvenile idiopathic arthritis (JIA) is a kind of autoimmune disorder which mainly injuring joint cartilage in children. However, various classification had been demonstrated among JIA patients suggesting a significant heterogeneity in disease pathogenesis. Moreover, it is still unknown that the differences of immune responses between JIA and other types of autoimmune diseases. Thus, the single cell RNA-sequencing (scRNA-seq) had been involved to explore the molecular mechanisms of JIA, and the regulating function of human leukocyte antigen-B27 (HLA-B27) in determining the heterogeneity in JIA. And we also analyzed the independent immune activity of JIA from other autoimmune diseases. We provided an immunological landscape of HLA-B27 positive and negative immune cells of JIA at scRNA-seq resolution. A unique proportion of CCR7^+^/RELB^+^/IRF1^+^ T cell had been identified in PBMCs of JIA as an independent responsor. And HLA-B27 enhanced the generation of CCR7^+^/RELB^+^/IRF1^+^ T cells. Functional analysis demonstrated that CCR7^+^/RELB^+^/IRF1^+^ T cells highly promoted the related gene expression of osteoclasts activation by producing IL-17, thus causing permanent damage to cartilage mainly in HLA-B27^+^ JIA. Our study provided evidence of cellular and molecular mechanisms involved in JIA pathogenesis and identified the critical roles for T cells inducing cartilage damages.

**Graphic Abstract**

**
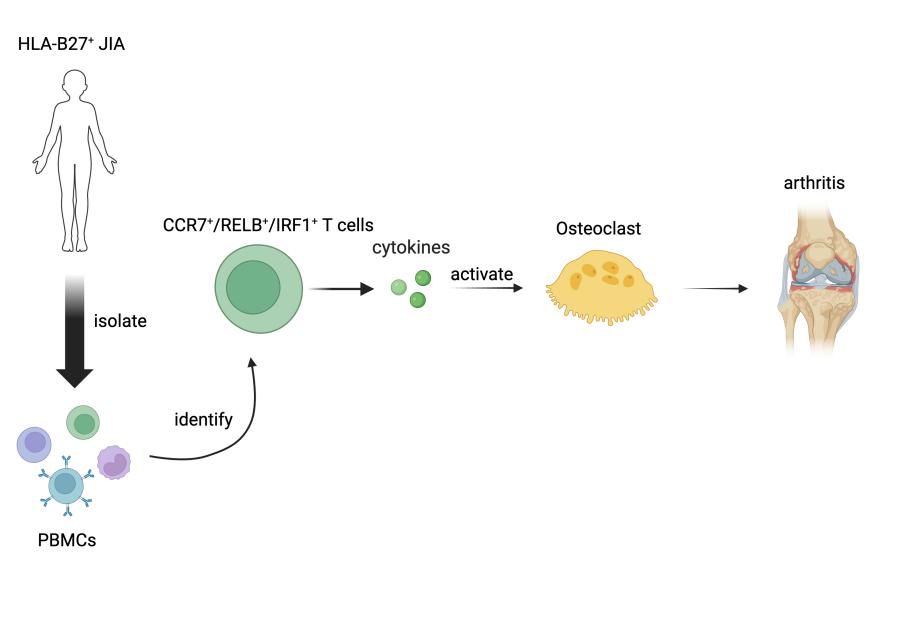
**

**Introduction**

Juvenile idiopathic arthritis (JIA) is a kind of rare autoimmune disease which had been considered only involved in children. Although cartilage degradation has been demonstrated as the dominant pathophysiological process in JIA. However, some severe comorbidities could also be observed with other system injuries. According to previous researches, the incidence of JIA varies among different populations, but the overall incidence remains around 7.8/100, 000 (1). It is difficult to be achieve a certain diagnosis of JIA at early stage (2). The structural and functional impairment of joints and bone damage had been found to be the major pathological changes in childhood (2, 3), definitely reducing the quality of life among affect population once irreversible cartilage or bone damages existing and then leaving a heavy burden in patients’ life. However, more than 50% of patients with JIA still suffer continuous inflammation activity in adulthood, resulting in long-term programmed diseases or inducing other autoimmune diseases (4). Thus, it is critical to demonstrate the etiology of JIA. It was reported that immune cells in peripheral blood had a clear role in mediating joint injury, but the pathogenesis in JIA had not yet been well understood. At present, most studies believe that JIA is caused by autoimmune dysfunction based on a combination of environmental and genetic factors(5). Besides, some studies demonstrated abnormal immune cells participated in arthritis as a major factor (6, 7). Previous researches attempted to demonstrate the association between inappropriate immune responses and JIA, involving abnormal molecular function of each immune cells and highly expressed cytokines. Due to the great limitation in determining the specific cellular subtypes of immune cells, it is rarely possible to explore the potential the heterogeneities of immune cell in JIA children compared to normal ones, which limits the essential molecular mechanisms in regulating JIA immune responses to be addressed.

Besides, there are several subtypes in JIA, which present various clinical outcomes and therapeutic strategies. HLA-B27 is a human leukocyte antigen (HLA) protein encoded by the HLA-B gene. It's a crucial part of the immune system. HLA-B27 is found on the surface of most cells in the body and plays a significant role in presenting protein fragments to immune cells called T lymphocytes. The HLA-B27 test is a key examination in JIA diagnosis, determining different classification and treatment. And enthesitis-associated arthritis (ERA) is a subtype of JIA and is considered as the most common JIA subtype in Asian. Current studies suggest that HLA-B27 contribute dominantly in inducing ERA, leading to more significant joint damage and cartilage degradation (8-11). However, it is still unknown that how HLA-B27 influences the immune cells developmental maturation and molecular function. Therefore, it is necessary to further elucidate the molecular mechanism of JIA pathogenesis under the influence of HLA-B27 and propose feasible therapeutic strategies targeting immune cells in JIA patients based on specific cellular and molecular features.

Currently, the single-cell RNA sequencing (scRNA-seq) made a great contribution in underline the heterogeneities among analyzed cells. It had been used to demonstrated different cellular function and differentiated strategies. ScRNA-seq had been used in studying the in the cellular characteristics among developmental, cancer, immune and cardiovascular research field. Importantly, the scRNA-seq could help to identify the inappropriate immune cellular subtypes and determining the mechanisms of such cells in mediating immune activities and responses. Herein, we applied scRNA-Seq to explore peripheral blood mononuclear cells (PBMCs) composition, proportion, gene expression characteristics, and developmental trajectory between JIA cases and healthy control. Moreover, we also explore the differences between the patients of positive and negative HLA-B27 test, to demonstrate the potential role of HLA-B27 in influencing T cell biological function. Finally, the scRNA-seq data of PBMCs of primary Sjögren's syndrome (pSS) and systemic lupus erythematosus (SLE) had been involved to validate the findings presented a high specificity in JIA, which was tried to identify the particular molecular function of JIA among other related autoimmune diseases.

**Results**

**The immunological landscape of JIA and cHC immune cells**

To investigate the differences in composition of immune cells between JIA patients and healthy controls, we performed scRNA-seq on PBMCs from JIA patients (JIA, n=7) and age-matched healthy children controls (cHC, n=3) (Fig 1A). After standard data preprocessing and quality control, we obtained a single-cell transcriptome of a total of 97,208 immune cells of PBMCs from JIA and cHC (Fig 1B). By examining the expression of the typical markers (*CD3E*, *CD79A*, *LYZ*, *CD14*, *FCGR3A*, *FCER1A*, *CD1C*, *NKG7*, *MZB1*, *PPBP*, *CD68* and *CYTL1*), we used an unsupervised graph-based clustering method, the immune cells identified in PBMCs include seven major cell types: B cells, T cells, Myeloid cells, NK, ILC, Neutrophil and Platelet (Fig 1C).

A total of six separated T cell subtypes had been identified from JIA and cHC by evaluating the expression of *CD3E*, *CD4*, *CD8A*, *CCR7*, *LEF1*, *SELL*, *TCF7*, *CD27*, *CD28*, *ICAM2*, *RGS1*, *CD69*, *GZMK*, *GZMA*, *CCL5*, *CD44*, *FOXP3*, *KLRB1* (Fig 1D, E), including CD4 Naïve (CD4 Naïve T cells), CD4 TCM (CD4^+^ central memory T cells), CD8 Naïve (CD8 Naïve T cells), CD8 TEM (CD8^+^ effector memory T cells), Treg (regulatory T cells) and CCR7^+^ T (CCR7 positive T cells) (Fig 1D, E). For B cells of JIA and cHC, three subtypes had been identified by evaluating the expression of *CD38*, *MZB1*, *XBP1*, *TNFRSF13B*, *CD79A*, *CD79B*, *CD19*, *MS4A1*, *IGHD*, *TCL1A* (Fig 1D, E), these included Naïve B (Naïve B cells), Memory B (Memory B cells) and Plasma B (Plasma B cells) (Fig 1D, E). And, there cellular subtypes had been identified among myeloid cells from JIA and cHC by evaluating the expression of *CD1C*, *FCER1A*, *CD68*, *LYZ*, *CD14*, *FCGR3A* (Fig 1D, E), as CD14 Mono (CD14^+^ Monocytes), Inter Mono (Inter Monocytes), CD16 Mono (CD16^+^ Monocytes) and DC (dendritic cells). The cell proportions of each cellular types of T cell, B cell and myeloid cell between JIA and cHC were taken in to initial comparison to demonstrate the most significant changed proportions in JIA (Fig 1F). Interestingly, the proportions of cellular types among T cells had been found with obvious heterogeneity between two groups. CD8 TEM and CCR7^+^ T cells compositions presented higher significant elevated ration among JIA cases compared to cHC ones. While the proportion of CD4 Naïve was significantly reduced in JIA (Fig 1F). However, there was no significant difference in the proportions of B cell subtypes and myeloid cell subtypes between JIA and cHC (Fig 1F).

Investigating molecular functional shifts within various immune cell types in JIA, we conducted statistical analyses on differentially expressed genes (DEGs) across diverse cellular categories between JIA and cHC (refer to Fig 1G). Our findings revealed that among T cell subtypes, CCR7^+^ T cells exhibited the highest count of DEGs (Fig 1G). Gene Ontology (GO) enrichment analysis of these DEGs originating from distinct immune cell types demonstrated that upregulated DEGs in JIA significantly enriched GO terms associated with immune function. Conversely, downregulated DEGs in JIA displayed notable enrichment in GO terms linked to growth and development (Fig 1G). Further exploration aimed at pinpointing the most responsive immune cell type to JIA involved the application of the Augur algorithm to our scRNA-Seq data. The analysis suggested that JIA potentially exerts the most pronounced influence on CCR7^+^ T cells compared to cHC (AUC > 0.9) (Fig 1H).

**CCR7+/RELB+/IRF1+ T cells contributed in the JIA as a dominant cluster**

The UMAP projection vividly illustrated the distribution of T cells across different patients with JIA and cHC (Fig S1A). Within the JIA and cHC datasets, there were 20,984 and 8,209 T cells, respectively. These T cells in both groups were categorized into six subtypes: CD4 Naïve, CD4 TCM, CD8 Naïve, CD8 TEM, Treg, and CCR7^+^ T cells (Fig 2A). Our analysis delved into the developmental trajectories of T cells in JIA and cHC, unveiling distinct differences. Notably, T cells in JIA exhibited fewer branching pathways during differentiation in contrast to those observed in cHC (Fig S1B). A heatmap depicting dynamic gene expression changes associated with T cell differentiation was constructed, clustering these genes into four distinct groups (Fig 2B). Within patients with JIA, the trajectory of T cell differentiation revealed an initial alteration in T cell homeostasis, followed by activation with enhanced expression of T cell receptors, culminating in the onset of a T cell-mediated inflammatory response (Fig 2B). Of significance during the differentiation of T cells in JIA, a notable emergence of CCR7^+^ T cells was observed at an early stage (Fig 2C, S2B). Conversely, in cHC individuals, the presence of CCR7^+^ T cells was limited during differentiation stages compared to those in JIA patients (Fig 2C, S2B).

To uncover the distinct mechanisms underlying the involvement of CCR7^+^ T cells in JIA, we separately analyzed their DEGs compared to CCR7^+^ T cells from cHC and CCR7^-^ T cells from JIA (Fig 2D). In comparison to CCR7^+^ T cells from cHC, 509 DEGs were upregulated in CCR7^+^ T cells from JIA, while 401 DEGs were upregulated in JIA's CCR7^+^ T cells versus other T cells from JIA (Fig 2D). Notably, the upregulated DEGs in both comparisons significantly enriched pathways related to Osteoclast differentiation (KEGG:04380) and T cell activation (GO:0042110) (Fig 2E). Within these DEGs, *RELB* and *IRF1* emerged as pivotal genes involved in osteoclast differentiation and T cell activation, respectively. Volcano plots highlighted the substantial differential expression and significance of *RELB* and *IRF1* in JIA's CCR7^+^ T cells compared to cHC's T cells (Fig 2F). Violin plots depicted distinct expression levels of *RELB* and *IRF1* genes across different T cell subtypes (Fig 2G), revealing significantly elevated expression in JIA's CCR7^+^ T cells compared not only to cHC's CCR7^+^ T cells but also to other T cells in JIA (Fig 2G). Isolation of CCR7+/RELB+/IRF1+ triple positive T cells from JIA patients and cHC individuals confirmed a presence in JIA's CCR7^+^ T cells, whereas cHC exhibited this triple positivity in subsets of CD4 Naïve and CD8 Naïve cells (Fig 2H, S2A). Importantly, the proportion of T cells exhibiting CCR7+/RELB+/IRF1+ triple positivity was significantly higher in JIA compared to cHC (Fig S2A). Further investigation of gene expression and functionality of CCR7+/RELB+/IRF1+ triple positive T cells in JIA versus other CCR7^+^ T cells revealed significantly enriched upregulated DEGs associated with cytokine production (GO:0001816), TNF signaling pathway (KEGG:04668), and IL-17 signaling pathway (KEGG:04657) (Fig 2I). Specifically, elevated expression levels of cytokine-related genes *NFKB1* and *NFKB2* were observed in JIA's CCR7^+^/RELB^+^/IRF1^+^ triple positive T cells compared to other CCR7^+^ T cells from JIA (Fig S3). This suggests a potential role for CCR7+/RELB+/IRF1+ triple positive T cells in JIA-induced bone damage through cytokine production.

**HLA-B27^+^ aggravated T cells mediating immune responses in JIA**

Though CCR7+/RELB+/IRF1+ T cells were observed notably in JIA patients, the molecular role of HLA-B27 in regulating T cell formation and its involvement in immune cell functionality maintenance remained unknown. Our JIA samples (n=7) comprised both HLA-B27^-^ (n=3) and HLA-B27^+^ (n=4) subtypes. Using UMAP projection, we visualized the distribution of T cells among patients with HLA-B27^+^ JIA and HLA-B27^-^ JIA (Fig S5A). Within HLA-B27^-^ JIA and HLA-B27^+^ JIA datasets, there were 7,891 and 13,093 T cells, respectively, divided into six subtypes (CD4 Naïve, CD4 TCM, CD8 Naïve, CD8 TEM, Treg, and CCR7^+^ T cell) (Fig 3A, S5A). Comparison of T cell subtype proportions between HLA-B27^+^ and HLA-B27^-^ JIA revealed a predominant presence of CCR7^+^ T cells in HLA-B27^+^ JIA, with minimal representation in HLA-B27^-^ JIA (Fig 3B). Consequently, CCR7+/RELB+/IRF1+ triple positive T cells were predominantly observed in HLA-B27^+^ JIA. Further investigation into the differentiation trajectories of T cells between HLA-B27^-^ and HLA-B27^+^ JIA (Fig S5B) revealed a substantial appearance of CCR7^+^ T cells in the early stages of T cell differentiation (Fig 3C, S5B). In contrast, HLA-B27^-^ JIA exhibited a limited presence of CCR7^+^ T cells during T cell differentiation compared to HLA-B27^+^ JIA (Fig 3C, S5B).

Heatmap analysis was conducted to explore dynamic gene expression changes associated with T cell transformation, categorizing genes into four clusters (Fig S5C). Notably, in HLA-B27^-^ JIA, T cells in both early and late stages of differentiation upregulated a greater number of genes associated with the immune response. Conversely, in HLA-B27^+^ JIA, T cells at the late stage of differentiation exhibited increased upregulation of immune response-related genes (Fig S5C).

To delve deeper into the gene expression variances among T cells in HLA-B27^+^ JIA and HLA-B27^-^ JIA, we examined the differential expression and functionality of upregulated Differentially Expressed Genes (DEGs) in T cells from both HLA-B27^+^ JIA and HLA-B27^-^ JIA compared to T cells from cHC. Notably, the upregulated DEGs in both HLA-B27^+^ and HLA-B27^-^ JIA prominently enriched pathways associated with T cell receptor (TCR) signaling (GO:0050852) and cytokine production (GO:0001816) (Fig 3D). However, differences emerged; cell communication (GO:0007154) exhibited higher enrichment in HLA-B27^-^ JIA T cells, whereas NF-kappaB complex (GO:0071159) showed significant enrichment solely in HLA-B27^+^ JIA T cells (Fig 3D). Further exploration focused on the expression levels of TCR-associated genes in HLA-B27^+^ JIA and HLA-B27^-^ JIA T cells compared to cHC (Fig 3E), revealing notably higher *EZR* expression in HLA-B27^+^ JIA T cells compared to HLA-B27^-^ JIA (Fig 3E).

Employing CellphoneDB, we investigated the interplay between T cells and other immune cell types in both HLA-B27^+^ JIA and HLA-B27^-^ JIA (Fig 3F). Heatmaps illustrating interaction intensity between different immune cell types revealed that CCR7^+^ T cells in both JIA subtypes, including CCR7+/RELB+/IRF1+ triple positive T cells, and CCR7^+^ T cells in cHC exhibited relatively weak interactions with other immune cell types (Fig 3F). Based on these interactions, we hypothesized that CCR7+/RELB+/IRF1+ triple positive T cells may not damage HLA-B27^+^ JIA cartilage through interactions with other immune cells.

To further discern the contrasting cytokine production capabilities of T cells in HLA-B27^+^ and HLA-B27^-^ JIA, we employed gene score profiles. Notably, T cells in HLA-B27^+^ JIA displayed elevated profile scores for IL-17 and TNF, while T cells in HLA-B27- JIA showcased higher profile scores for IL-2 and NF-kappaB (Fig 3G). We presented violin plots illustrating the expression patterns of 10 representative genes, including *IL17A*, *IL2*, *TNF*, *IFNγ*, *NFKB1*, *NFKB2*, *IL18*, *IL4*, *IL6*, and *IL1B* (Fig 3H, S5). Among these, *TNF*, *NFKB1*, and *NFKB2* exhibited predominant expression in T cells of HLA-B27^+^ JIA. Meanwhile, the expression of *IFNγ* did not significantly differ between T cells of HLA-B27^+^ JIA and HLA-B27^-^ JIA (Fig 3H, S5).

**CCR7+/RELB+/IRF1+ T cells independently response for JIA**

To discern differences in immune cell compositions across various autoimmune diseases, including JIA, pSS, and SLE, we opted to analyze PBMCs obtained from primary Sjögren's syndrome (pSS, n=5), systemic lupus erythematosus (SLE, n=4), and adult healthy controls (aHC, n=5), in conjunction with PBMCs from JIA and cHC. Following data preprocessing and quality checks, we acquired a total of 137,454 single-cell transcriptomes of immune cells (Fig S6A). The UMAP projection visually represented the distribution of immune cells (Fig 4A, S6A). Distinguishing from JIA and cHC, we identified three additional T cell subtypes—CD4 TEM (CD4^+^ Effector Memory T cells), MAIT (Mucosal-associated invariant T cells), and PTPRC^+^ T (PTPRC^+^ T cells)—in pSS, SLE, and aHC (Fig 4A, S6B). Comparing cell subtype proportions of T cells, B cells, and Myeloid cells among patients with different diseases and healthy controls revealed the exclusive presence of CCR7^+^ T cells in patients with JIA and cHC (Fig 4A, S7A, B). Further analysis aimed to pinpoint the immune cell types most responsive to JIA, pSS, and SLE involved employing the Augur algorithm on scRNA-Seq data, focusing on the shared immune cells among JIA, pSS, and SLE (Fig 4B). Our findings indicated that pSS potentially exerts a significant impact on CD4 Naïve and CD8 Naïve cells compared to aHC (AUC > 0.8). Conversely, SLE demonstrated a notable impact on DC and CD16 Mono cells compared to aHC (AUC > 0.96) (Fig 4B).

As CD4 Naïve, CD4 TCM, CD8 Naïve, and CD8 TEM were shared T cell subtypes among patients with JIA, pSS, and SLE, we analyzed the upregulated DEGs specific to these four T cell subtypes, comparing JIA with cHC, pSS with aHC, and SLE with aHC, respectively (Fig 4C). Notably, both pSS and SLE exhibited a higher count of upregulated DEGs compared to JIA (Fig 4C). Subsequently, we conducted GO terms and KEGG pathways enrichments based on the upregulated DEGs among CD4 Naïve, CD4 TCM, CD8 Naïve, CD8 TEM, and all T cells within different autoimmune diseases (Fig 4D). Remarkably, the TCR signaling pathway (GO:0050852) and cytokine production (GO:0001816) were significantly enriched among upregulated DEGs in JIA, pSS, and SLE T cells (Fig 4D). A heatmap analysis revealed increased TCR signaling pathway-related upregulated DEGs in both JIA and pSS (Fig 4E). Moreover, the expression level of *EZR* in JIA T cells significantly surpassed that in pSS and SLE (Fig 4E). Exploration of *RELB* and *IRF1* expression on T cells from JIA, pSS, and SLE revealed lower levels in pSS and SLE T cells compared to aHC (Fig S7C). Across the T cell populations of these autoimmune diseases, *CCR7*, *RELB*, and *IRF1* were notably highly expressed in JIA T cells (Fig 4F), which revealed that CCR7+/RELB+/IRF1+ T cells independently response for JIA. Additionally, a preliminary analysis of immune cell interactions among patients with these autoimmune diseases revealed minimal interaction between T cell subtypes and other immune cell types (Fig 4G).

We employed gene score profiles to delve deeper into the differences in cytokine production abilities of T cells in JIA compared to those in pSS and SLE. The results revealed that both T cells and CD4 T cells in JIA exhibited a potentially heightened capacity to produce IL-17, while T cells, especially CD4 T cells, in pSS displayed increased potential for IL-2, TNF, and NF-kappaB production (Fig 4H, S7B, S7C). Subsequently, we calculated shared and non-shared upregulated DEGs in T cells and CD4 T cells among JIA, pSS, and SLE (Fig S7A). Their capacity to produce IFN-β, IFN-γ, and NF-kappaB was compared by examining the upregulated DEGs in T cells across the three autoimmune diseases. Notably, T cells in JIA, pSS, and SLE exhibited robust capability in producing NF-kappaB, IFN-γ, and IFN-β (Fig 4I).

**Characteristics of B cells between HLA-B27^-^ and HLA-B27^+^ JIA**

The UMAP projection illustrated the distribution of B cell subtypes among various patients with JIA, including HLA-B27^+^ JIA, HLA-B27^-^ JIA, and cHC (Fig 5A). Notably, HLA-B27^+^ JIA, HLA-B27^-^ JIA, and cHC encompassed 3,635, 2,134, and 4,473 B cells, respectively (Fig 5A). Within JIA and cHC, B cells were categorized into three subtypes: Naïve B, Memory B, and Plasma B (Fig S9A). The differentiation trajectories of B cells from JIA exhibited no anomalies (Fig S9B). Further subdivision of B cells in HLA-B27^+^ JIA and HLA-B27^-^ JIA also revealed three subtypes: Naïve B, Memory B, and Plasma B (Fig S9C). Comparing the differentiation trajectories of B cells between HLA-B27^-^ JIA and HLA-B27^+^ JIA unveiled significant disparities. Notably, Plasma B cells in HLA-B27^-^ JIA emerged early in development, whereas in HLA-B27^+^ JIA, they appeared late in development (Fig 5B, S9D). To investigate gene expression dynamics associated with B cell transformation, we segmented these genes into four clusters and constructed heatmaps (Fig 5C). At the early stage of differentiation, Plasma B cells in HLA-B27^-^ JIA showed increased upregulation of genes related to the immune response. In contrast, B cells in HLA-B27^+^ JIA, during both early and late differentiation stages, displayed elevated expression of genes associated with the immune response (Fig 5C).

We conducted a comprehensive analysis of upregulated DEGs in B cell subtypes from both HLA-B27^-^ JIA and HLA-B27^+^ JIA compared to cHC (Fig 5D). These DEGs showed significant enrichment in immune-related GO terms, including immune system processes (GO:0002376), immune response (GO:0006955), and B cell activation (GO:0042113) (Fig 5E). Notably, DEGs exclusively upregulated in Naïve B of both HLA-B27^-^ JIA (n=178, 49.3%) and HLA-B27^+^ JIA (n=104, 28.8%), as well as those co-upregulated by Naïve B in both conditions (n=79, 21.9%), exhibited significant enrichment in MHC class II protein complex (GO:0042613) (Fig 5D, E). Furthermore, DEGs co-upregulated by Memory B in HLA-B27^-^ JIA and HLA-B27^+^ JIA (n=271, 47.7%) were notably enriched in the B cell receptor signaling pathway (GO:0050853) (Fig 5D, E). We visualized the gene expression of the B cell receptor signaling pathway using a heatmap, revealing slightly more B cell receptor signaling-associated DEGs in Memory B from HLA-B27^-^ JIA than in those from HLA-B27^+^ JIA, while overall gene expression profiles appeared similar (Fig 5F). Noteworthy differences emerged in the expression levels of specific genes: Naïve B of HLA-B27^-^ JIA showed elevated levels of *HLA-DMB*, *HLA-DOB*, and *B2M*, whereas Naïve B of HLA-B27^+^ JIA displayed increased expression of *HLA-DPB1*, *HLA-DQA2*, and *HLA-DQB1* (Fig 5G). Additionally, both Naïve B populations in HLA-B27^-^ JIA and HLA-B27^+^ JIA exhibited significantly heightened expression levels of *HLA-DRA*, *HLA-DRB1*, and *CD74* (Fig 5G).

**Characteristics of Myeloid cells between HLA-B27^-^ and HLA-B27^+^ JIA**

The UMAP projection illustrated the distribution of Myeloid cell subtypes among patients with JIA, encompassing HLA-B27^+^ JIA, HLA-B27^-^ JIA, and cHC (Fig 6A). Notably, HLA-B27^+^ JIA, HLA-B27^-^ JIA, and cHC consisted of 4,889, 4,082, and 3,354 Myeloid cells, respectively (Fig 6A). An analysis comparing the differentiation trajectories of monocytes in JIA versus cHC revealed minimal divergence between them (Fig S10A). Further exploration of the differentiation trajectories of monocytes between HLA-B27^-^ JIA and HLA-B27^+^ JIA highlighted distinct patterns (Fig 6B, S10B). Notably, CD16 Mono in HLA-B27^-^ JIA emerged early in differentiation, while Inter Mono appeared later in HLA-B27^-^ JIA, contrasting with early emergence in HLA-B27^+^ JIA (Fig 6B, S10B). To understand the gene expression dynamics associated with Monocyte transformation, we categorized these genes into four clusters and visualized them using heatmaps (Fig S10C). Interestingly, CD14 Mono and CD16 Mono in HLA-B27^-^ JIA exhibited increased upregulation of genes related to the immune response at the early stage of differentiation, whereas in HLA-B27^+^ JIA, these cell types showed greater upregulation of immune response-related genes at the middle stage of differentiation (Fig S10C).

The UMAP projection illustrated the distribution of Myeloid cell subtypes among patients with JIA, encompassing HLA-B27^+^ JIA, HLA-B27^-^ JIA, and cHC (Fig 6A). Notably, HLA-B27^+^ JIA, HLA-B27^-^ JIA, and cHC consisted of 4,889, 4,082, and 3,354 Myeloid cells, respectively (Fig 6A). An analysis comparing the differentiation trajectories of monocytes in JIA versus cHC revealed minimal divergence between them (Fig S10A). Further exploration of the differentiation trajectories of monocytes between HLA-B27^-^ JIA and HLA-B27^+^ JIA highlighted distinct patterns (Fig 6B, S10B). Notably, CD16 Mono in HLA-B27^-^ JIA emerged early in differentiation, while Inter Mono appeared later in HLA-B27^-^ JIA, contrasting with early emergence in HLA-B27^+^ JIA (Fig 6B, S10B). To understand the gene expression dynamics associated with Monocyte transformation, we categorized these genes into four clusters and visualized them using heatmaps (Fig S10C). Interestingly, CD14 Mono and CD16 Mono in HLA-B27^-^ JIA exhibited increased upregulation of genes related to the immune response at the early stage of differentiation, whereas in HLA-B27^+^ JIA, these cell types showed greater upregulation of immune response-related genes at the middle stage of differentiation (Fig S10C).

We conducted an analysis of upregulated DEGs in all Myeloid cell subtypes of HLA-B27^-^ JIA and HLA-B27^+^ JIA compared to cHC (Fig 6C). Specifically, the upregulated DEGs in CD14 Mono and CD16 Mono from both HLA-B27^-^ JIA and HLA-B27^+^ JIA demonstrated significant enrichment in immune-related GO terms, including immune system processes (GO:0002376), immune response (GO:0006955), and MAPK cascade (GO:0007255) (Fig 6D). Further GO enrichment analysis revealed that DEGs co-upregulated by CD14 Mono in both HLA-B27^-^ JIA and HLA-B27^+^ JIA (n=133, 30.2%) were notably enriched in IL-1 beta production (GO:0050720), IL-6 production (GO:0042226), and IL-8 production (GO:0042228). Intriguingly, DEGs exclusively upregulated in CD14 Mono of HLA-B27- JIA (n=190, 43.2%) also displayed significant enrichment in IL-6 production (GO:0042226) and IL-8 production (GO:0042228) (Fig 6E, F). Subsequent comparison of the IL production capacity of CD14 Mono in HLA-B27^-^ JIA and HLA-B27^+^ JIA through heatmap and Sankey diagram analysis revealed that CD14 Mono in both conditions produced IL-1β, IL-6, and IL-8. Notably, HLA-B27- JIA demonstrated a higher number of upregulated DEGs associated with IL-6 production (GO:0042226) and IL-8 production (GO:0042228), indicating a potentially heightened capacity for pro-inflammatory interleukin production in CD14 Mono from HLA-B27^-^ JIA (Fig 6E, F).

We delved into the DEGs and GO enrichment results focused on CD16 Mono in both HLA-B27^-^ JIA and HLA-B27^+^ JIA. Notably, the DEGs co-upregulated by CD16 Mono in both conditions (n=324, 45.7%) displayed significant enrichment in IL-1 beta production (GO:0050720), IL-6 production (GO:0042226), and IL-8 production (GO:0042228). Additionally, DEGs exclusively upregulated in CD16 Mono of HLA-B27^-^ JIA (n=295, 41.6%) showed notable enrichment in IL-8 production (GO:0042228) (Fig 6C, D). Further comparison of the interleukin production capacity of CD16 Mono between HLA-B27^-^ JIA and HLA-B27^+^ JIA through heatmap and Sankey diagram analysis revealed that both subsets produced IL-1β, IL-6, and IL-8. Intriguingly, HLA-B27^-^ JIA displayed a higher count of upregulated DEGs associated with IL-8 production (GO:0042228), suggesting a potentially heightened ability for pro-inflammatory IL production in CD16 Mono from HLA-B27^-^ JIA (Fig 6E, F).

**Discussion**

JIA is a kind of autoimmune condition emerging before 16 years of age (5, 12). And HLA-B27, had been considered to be participated in the prognosis of JIA (13, 14). In JIA, HLA-B27 is acknowledged as a risk factor implicated in exacerbating arthritis severity(15). However, not all individuals with HLA-B27 develop JIA, suggesting involvement of other genetic or environmental factors, and the delineating JIA into distinct clinical subtypes solely based on HLA-B27 status remains uncertain (16, 17). The bone loss observed in JIA often results in decreased bone mineral density (BMD) (3, 18), offering insights into JIA's etiology and pathogenesis. The immune system's aberrant response, involving T cells, B cells, and cytokine dysregulation, is increasingly recognized as a central mechanism in JIA pathogenesis. Dysregulated immune cells contribute to chronic inflammation and tissue damage, particularly in the joints, leading to the characteristic arthritis seen in JIA patients. Further insights into the cellular and molecular mechanisms through advanced techniques like scRNA-seq have revealed specific subsets of T cells, such as CCR7+/RELB+/IRF1+ triple positive T cells, associated with disease activity and bone damage in JIA, especially in the context of HLA-B27 positivity. To further elucidate JIA's immune cell characteristics, we conducted a novel investigation utilizing single-cell RNA sequencing. Our study delved into JIA's pathogenesis, revealing a pivotal link between differential gene expression in T cells and bone damage, particularly in HLA-B27^+^ JIA. Notably, the activation of CCR7+/RELB+/IRF1+ triple positive T cells was found to instigate and facilitate osteoclast differentiation. Furthermore, we conducted a comparative analysis of T cell functionality in JIA alongside two other autoimmune diseases, pSS, and SLE.

We initially compared the distribution of immune cell subtypes between JIA and cHC, noticing a notably higher ratio of CCR7^+^ T cells within JIA's T cell population compared to cHC. Unlike cHC, JIA's T cells exhibited an increased presence of CCR7^+^ T cells early in their differentiation process. This observation led us to hypothesize the pivotal role of CCR7^+^ T cells in JIA's pathogenesis. To validate this hypothesis, we examined the DEGs within CCR7^+^ T cells from JIA versus CCR7^+^ T cells from cHC and other T cells within JIA. The upregulated DEGs within CCR7^+^ T cells from JIA were notably associated with T cell activation and osteoclast differentiation (19, 20). T cells are instrumental in initiating immune responses, and their overactivation frequently underlies autoimmune diseases (21, 22). Moreover, cytokines derived from activated T cells have been linked to promoting osteoclast differentiation (23, 24). We delved deeper into the significantly upregulated DEGs within CCR7^+^ T cells from JIA. *RELB* has a known role in osteoclast generation, while IRF1's involvement in abnormal T cell activation contributes to various autoimmune conditions (25-28). Analyzing the upregulated DEGs by CCR7+/RELB+/IRF1+ triple positive T cells in JIA compared to other CCR7^+^ T cells revealed enrichment in the IL-17 and TNF signaling pathways. Both IL-17 and TNF are pivotal in autoimmune diseases like JIA and ankylosing spondylitis (29, 30). IL-17's direct promotion of osteoclast formation has been evident in rheumatoid arthritis models (31, 32). Consequently, our findings suggest a potential mechanism wherein CCR7+/RELB+/IRF1+ triple positive T cells might induce osteoclast differentiation via cytokine production, particularly IL-17, contributing to bone damage in JIA patients. Further exploration is warranted to fully elucidate this pathway's intricacies and potential therapeutic targets.

Following the categorization of JIA into HLA-B27^+^ and HLA-B27^-^ subsets, a notable prevalence of CCR7^+^ T cells was observed in JIA. HLA-B27 is closely linked to JIA, especially in boys, where its presence is associated with clinical sacroiliitis symptoms and impedes symptom remission (11, 33, 34). Among the genes involved in the TCR signaling pathway, increased expression of *EZR* was noticed in T cells of HLA-B27^+^ JIA. This encoded ezrin protein is known to interact with CD44, a significant contributor to osteoarthritis progression (35, 36). When comparing cytokine production between T cells in HLA-B27^+^ JIA and HLA-B27^-^ JIA, a higher capacity for IL-17 and TNF production was evident in T cells from HLA-B27^+^ JIA. Based on these findings, the upregulation of EZR expression by CCR7+/RELB+/IRF1+ triple positive T cells was observed. This potentially augments the interaction between CD44 and chondrocytes, known to produce substantial IL-17 amounts. Consequently, this cascade might trigger chondrocyte damage in JIA, especially within the context of HLA-B27^+^ JIA.

Then, we illustrated the characterization and gene expression of T cells in pSS and SLE, comparing them with those in JIA. Notably, CCR7^+^ T cells were absent in both pSS and SLE. Specific genes—*CCR7*, *RELB*, and *IRF1*—displayed heightened expression exclusively in T cells from JIA. Moreover, *EZR* expression levels were notably higher in T cells from JIA compared to those in pSS and SLE. T cells within JIA, including CD4 T cells, exhibited a greater capacity for IL-17 production than their counterparts in pSS and SLE. From these observations, we conjectured that the gene expression and functionality of CCR7+/RELB+/IRF1+ triple positive T cells could represent a distinctive feature of JIA, setting it apart from pSS and SLE and potentially serving as a therapeutic target for JIA.

When BCR aggregation triggers signaling, B cells undertake the internalization, processing, and presentation of bound antigens via the surface Major MHC Class II molecules (37). *CD74* functions as an MHC-II chaperone, regulating its presence on the cell surface (38). Genetic links have tied *HLA-DRB1* and *HLA-DPB1* to JIA predisposition (39). HLA-DM's role involves stabilizing the peptide-receptive state of MHC-II through transient association, facilitating the removal of weakly bound peptides and loading high-affinity peptides, including many immunodominant epitopes, onto B cell surfaces (40). Our investigation revealed distinct gene expression patterns associated with MHC-II antigen processing and presentation in Naïve B cells between HLA-B27^-^ JIA and HLA-B27^+^ JIA. These differences in gene expression might play a role in the pathogenesis of the two JIA subtypes. BCR signaling significantly influences B cell-mediated autoimmune inflammation, suggesting that inhibiting BCR signaling could emerge as a novel treatment approach for autoimmune diseases (41). Our findings indicated heightened BCR signaling in Memory B cells in both HLA-B27^-^ JIA and HLA-B27^+^ JIA, showcasing similar gene expression profiles. We postulate that the development of inhibitors targeting BCR signaling pathways might offer potential therapeutic avenues for JIA treatment.

Monocytes, known for their diverse immunomodulatory functions, actively contribute to autoimmune diseases by regulating inflammation and tissue repair (42). CD14 Mono, specifically, are recognized for their production of potent pro-inflammatory interleukins such as IL-1β and IL-6, crucial in supporting inflammation (43, 44). Among the IL-1 family, IL-1β, a prominent product of myeloid cells, has been extensively studied and demonstrated to have elevated levels in arthritis (45, 46). IL-6, abundantly present in the synovial fluid of RA patients, plays a pivotal role in various chronic inflammatory and autoimmune diseases (47). Research by Atsuto Naruke et al. highlighted the significance of IL-1β-mediated IL-8 expression in autoimmune disease pathogenesis (48). In our investigation, CD14 Mono in both HLA-B27^-^ JIA and HLA-B27^+^ JIA exhibited the capability to produce three pro-inflammatory interleukins (IL-1β, IL-6, and IL-8). Targeting CD14 Mono to inhibit IL-6 and IL-8 might offer therapeutic potential for treating HLA-B27^-^ JIA, especially considering the stronger IL-6 and IL-8 production observed in this subtype. CD16 Mono, often regarded as pro-inflammatory cells due to their association with disease mobilization and secretion of crucial inflammatory cytokines, were found in both HLA-B27^-^ JIA and HLA-B27^+^ JIA, demonstrating the ability to produce IL-1β, IL-6, and IL-8 (49). Notably, IL-8 production appeared stronger in patients with HLA-B27^-^ JIA.

In summary, this study provided a transcriptional landscape of immune cells in JIA patients at single cell resolution. The results initially identified that cluster of CCR7^+^/RELB^+^/IRF1^+^ T cells contributed dominantly in mediating the pathogenesis of JIA, leading to bone degradation by osteoclast overactivation. Moreover, the HLA-B27 aggregated the immune activity of CCR7^+^/RELB^+^/IRF1^+^ T cells, resulting in adverse prognosis for JIA individuals with HLA-B27^+^. Also, CCR7^+^/RELB^+^/IRF1^+^ T cells had been revealed as an independent responsor in JIA among other types of autoimmune diseases, severing as potential therapeutic target.

**Methods**

**Sample preparation for 10x Genomics**

Sex was not considered as a biological variable, and both female and male participates had been involved in the research. Seven children were diagnosed as JIA, three patients with HLA-B27- and four patients with HLA-B27+. Three age-matched volunteers were enrolled as children healthy control (cHC) were collected. Patients with autoimmune disease before JIA onset, monoclonal antibody therapy, or other blood diseases were excluded. Participants’ parents or guardians provided informed consent to participate in this study. The Ethics Committee approved the study of Sichuan University and West China Second Hospital, China. The published data on scRNA-seq of PBMCs of 5 patients with pSS (pSS) (GSE157278) (50), 4 patients with SLE (SLE) (GSE137029) (51) and 5 adult healthy control ones (aHC) (GSE157278) (50) had been reanalyzed.

**Single cell collection and 10x Genomics single-cell mRNA sequencing**

Peripheral blood samples (4 mL each sample) were collected from the ten subjects. The single-cell suspensions of scRNA-seq samples were converted to barcoded scRNA-seq libraries using the Chromium Single Cell 5′ Library, Gel Bead and Multiplex Kit, and Chip Kit (10x Genomics). The Chromium Single Cell 5′ v2 Reagent (10x Genomics) kit was used to prepare single-cell RNA libraries according to the manufacturer's instructions.

**Single-cell RNA data alignment and quality control**

The raw 10x Genomics sequencing data were processed using CellRanger v6.1.2 (52), and the 10x human transcriptome GRCh38-2020-A was used as the reference genome. Single-cell read counts from all samples were converted to a Seurat object using the Seurat (v4.2.0) (53) analysis package in R (v4.2.1). For Seurat object, we filtered the data based on the unique molecular identifiers (UMIs) and the number of detected genes. The cells with > 1500 UMIs, > 1000 detected genes and fewer than 10% of read from mitochondrial genes were preserved. The doublets were identified using DoubletFinder (v2.0.3) (54) package in R.

**Data dimension reduction and cell type annotation**

The Seurat data were normalized using Seurat’s NormalizeData function. Highly variable genes were detected using Seurat’s FindVariableFeatures function. The data were further scaled using Seurat’s ScaleData function. We then completed the Principle component analysis (PCA). The cells’ clusters were identified using Uniform Manifold Approximation and Projection (UMAP). We had divided the cells into seven types (B cells, T cells, Myeloid cells, NK, ILC, Neutrophil and Platelet) according to the classical marker genes (Figure 6B). We used the calculate_auc function in Augur to determine the priority of cell types by comparing JIA, pSS and SLE with corresponding healthy controls, respectively (55).

**Sub-clustering of T cells, B cells and myeloid cells**

T cells, B cells and myeloid cells were extracted from PBMC for further sub-clustering. PCA and clustering were also performed as described in Data dimension reduction and cell type annotation.

**Detection of single-cell level differentially expressed genes and enrichment analysis**

Seurat’s FindMarkers function was used to identify the differentially expressed genes (DEGs) between the disease group and the control group of the same cell type. DEGs were required to average log2 fold change >0.25 and adjusted p-value < 0.05. The GO and KEGG enrichment analysis of the DEGs was implemented using g:Profiler (https://biit.cs.ut.ee/gprofiler/gost) (56).

**Cell trajectory analysis**

The Monocle (v2.26.0) R package (57) was used to infer potential cell lineage trajectories between different cell types. We inferred and characterized the lineage trajectories of T cells, B cells and monocytes at the single-cell level. The UMI count matrices for each major cell type were used as input. We followed the Monocle2 tutorial to use the newCellDataSet function to create a CellDataSet object with the parameter expressionFamily = negbinomial.size(). Based on the significant DEGs, we used DDRTree algorithm to reduce the dimension of data. Cell lineage trajectories based on cell types were inferred by dimensionality reduction and cell ordering using default parameters of Monocle2 and visualized using the plot_cell_trajectory function. According to the cell trajectories, the differentialGeneTest function was used to find genes along to the pseudotime direction. We then visualized the heatmap of significant pseodutime-dependent genes.

**Module scores for gene sets expression**

AddModuleScore from Seurat (v4.3.0.1) was used to calculate the average expression level of each cell type or group at the individual cell level, minus the total expression of the control gene set.

**Cell-cell interaction analysis**

To comprehensively analyze the cell-cell interactions between immune cells in JIA, pSS and SLE, we used CellPhoneDB (v4.0.0) (58). We identified potential ligand-receptor interactions based on the expression of receptors by one subset of cells and ligands by another. Normalized counts of JIA (HLA-B27^-^ and HLA-B27^+^), cHC, pSS and SLE were obtained as input data for the CellPhoneDB algorithm.

**Data visualization**

All figures were generated using ggplot2 (v3.4.1), pheatmap (v1.0.12), plotly (v4.10.3), volcano3D (v2.0.9), ggvenn (v0.1.10) and ggalluvial (v0.12.5) in R (v4.2.1). Violin plots were defined as follows: the Wilcoxon rank-sum test tested the median means of continuous variables in two groups.

**Study approval**

Patient data were collected by Yifei Li. And this study was approved by the Ethics Committee of West China Second Hospital of Sichuan University (2021-069). For the participants, we obtained the written informed consent to participate in this research from all the reported patients’ parents for the inclusion of the patients’ clinical details in subsequent publications.

Data availability

All the sequencing data has been deposited at GSA (<https://ngdc.cncb.ac.cn/gsub/>, HRA006261, and reviewers could access these data from the link https://ngdc.cncb.ac.cn/gsa-human/browse/HRA006261). Other data sets used and analyzed in this study are available upon reasonable requests.

Author contributions

Yifei Li, ZF and JG conceptualized the study; LH, XG, HG, KZ, Yue Lan, ML, JG, ZF and Yifei Li designed the analysis; HG, XL, YH and Yifei Li were the physicians of the involved patients; LH, XG, Yue Lan, ML and ZF analyzed data; LH, XG, JG, ZF and Yifei Li wrote or edited the manuscript. Order of co–first authors was determined based on the amount of work contributed.

Acknowledgements

YL was funded by National Natural Science Foundation of China (grant no. 82270249).

**References**

1. Cross M, Smith E, Hoy D, Carmona L, Wolfe F, Vos T, et al. The global burden of rheumatoid arthritis: estimates from the global burden of disease 2010 study. *Ann Rheum Dis.* 2014;73(7):1316-22.

2. Bansal N, Pasricha C, Kumari P, Jangra S, Kaur R, and Singh R. A comprehensive overview of juvenile idiopathic arthritis: From pathophysiology to management. *Autoimmunity reviews.* 2023;22(7):103337.

3. Martini A, Lovell DJ, Albani S, Brunner HI, Hyrich KL, Thompson SD, et al. Juvenile idiopathic arthritis. *Nature reviews Disease primers.* 2022;8(1):5.

4. Tollisen A, Selvaag AM, Aulie HA, Lilleby V, Aasland A, Lerdal A, et al. Physical Functioning, Pain, and Health-Related Quality of Life in Adults With Juvenile Idiopathic Arthritis: A Longitudinal 30-Year Followup Study. *Arthritis care & research.* 2018;70(5):741-9.

5. Prakken B, Albani S, and Martini A. Juvenile idiopathic arthritis. *Lancet (London, England).* 2011;377(9783):2138-49.

6. Bessis N, Decker P, Assier E, Semerano L, and Boissier MC. Arthritis models: usefulness and interpretation. *Semin Immunopathol.* 2017;39(4):469-86.

7. Woodell-May JE, and Sommerfeld SD. Role of Inflammation and the Immune System in the Progression of Osteoarthritis. *J Orthop Res.* 2020;38(2):253-7.

8. Adrovic A, Barut K, Sahin S, and Kasapcopur O. Juvenile Spondyloarthropathies. *Curr Rheumatol Rep.* 2016;18(8):55.

9. Flatø B, Hoffmann-Vold AM, Reiff A, Førre Ø, Lien G, and Vinje O. Long-term outcome and prognostic factors in enthesitis-related arthritis: a case-control study. *Arthritis Rheum.* 2006;54(11):3573-82.

10. Bryan AR, and Rabinovich CE. Enthesitis-related arthritis: time to re-define? *Curr Rheumatol Rep.* 2014;16(12):466.

11. Berntson L, Nordal E, Aalto K, Peltoniemi S, Herlin T, Zak M, et al. HLA-B27 predicts a more chronic disease course in an 8-year followup cohort of patients with juvenile idiopathic arthritis. *The Journal of rheumatology.* 2013;40(5):725-31.

12. Zaripova LN, Midgley A, Christmas SE, Beresford MW, Baildam EM, and Oldershaw RA. Juvenile idiopathic arthritis: from aetiopathogenesis to therapeutic approaches. *Pediatric rheumatology online journal.* 2021;19(1):135.

13. Chen B, Li J, He C, Li D, Tong W, Zou Y, et al. Role of HLA-B27 in the pathogenesis of ankylosing spondylitis (Review). *Molecular medicine reports.* 2017;15(4):1943-51.

14. Sorrentino R, Böckmann RA, and Fiorillo MT. HLA-B27 and antigen presentation: at the crossroads between immune defense and autoimmunity. *Molecular immunology.* 2014;57(1):22-7.

15. Żuber Z, Turowska-Heydel D, Sobczyk M, and Chudek J. Prevalence of HLA-B27 antigen in patients with juvenile idiopathic arthritis. *Reumatologia.* 2015;53(3):125-30.

16. Schiellerup P, Krogfelt KA, and Locht H. A comparison of self-reported joint symptoms following infection with different enteric pathogens: effect of HLA-B27. *The Journal of rheumatology.* 2008;35(3):480-7.

17. Classification Criteria for Spondyloarthritis/HLA-B27-Associated Anterior Uveitis. *American journal of ophthalmology.* 2021;228:117-25.

18. Brabnikova Maresova K, Jarosova K, Pavelka K, and Stepan JJ. Bone status in adults with early-onset juvenile idiopathic arthritis following 1-year anti-TNFα therapy and discontinuation of glucocorticoids. *Rheumatology international.* 2013;33(8):2001-7.

19. Gorentla BK, and Zhong XP. T cell Receptor Signal Transduction in T lymphocytes. *Journal of clinical & cellular immunology.* 2012;2012(Suppl 12):5.

20. Shah K, Al-Haidari A, Sun J, and Kazi JU. T cell receptor (TCR) signaling in health and disease. *Signal transduction and targeted therapy.* 2021;6(1):412.

21. Rosetti F, Madera-Salcedo IK, Rodríguez-Rodríguez N, and Crispín JC. Regulation of activated T cell survival in rheumatic autoimmune diseases. *Nature reviews Rheumatology.* 2022;18(4):232-44.

22. Sun Y, Zhu X, Chen X, Liu H, Xu Y, Chu Y, et al. The mediator subunit Med23 contributes to controlling T-cell activation and prevents autoimmunity. *Nature communications.* 2014;5:5225.

23. Karieb S, and Fox SW. Suppression of T cell-induced osteoclast formation. *Biochemical and biophysical research communications.* 2013;436(4):619-24.

24. Fischer V, and Haffner-Luntzer M. Interaction between bone and immune cells: Implications for postmenopausal osteoporosis. *Seminars in cell & developmental biology.* 2022;123:14-21.

25. Zhao Z, Hou X, Yin X, Li Y, Duan R, Boyce BF, et al. TNF Induction of NF-κB RelB Enhances RANKL-Induced Osteoclastogenesis by Promoting Inflammatory Macrophage Differentiation but also Limits It through Suppression of NFATc1 Expression. *PloS one.* 2015;10(8):e0135728.

26. Vaira S, Johnson T, Hirbe AC, Alhawagri M, Anwisye I, Sammut B, et al. RelB is the NF-kappaB subunit downstream of NIK responsible for osteoclast differentiation. *Proceedings of the National Academy of Sciences of the United States of America.* 2008;105(10):3897-902.

27. Chen J, Peng L, Zhao Z, Yang Q, Yin F, Liu M, et al. HDAC1 potentiates CD4 + T cell activation by inhibiting miR-124 and promoting IRF1 in systemic lupus erythematosus. *Cellular immunology.* 2021;362:104284.

28. Zhang Z, Shi L, Song L, Ephrem E, Petri M, and Sullivan KE. Interferon regulatory factor 1 marks activated genes and can induce target gene expression in systemic lupus erythematosus. *Arthritis & rheumatology (Hoboken, NJ).* 2015;67(3):785-96.

29. Kostik MM, Makhova MA, Maletin AS, Magomedova SM, Sorokina LS, Tsukasaki M, et al. Cytokine profile in patients with chronic non-bacterial osteomyelitis, juvenile idiopathic arthritis, and insulin-dependent diabetes mellitus. *Cytokine.* 2021;143:155521.

30. Ahluwalia B, Moraes L, Magnusson MK, and Öhman L. Immunopathogenesis of inflammatory bowel disease and mechanisms of biological therapies. *Scandinavian journal of gastroenterology.* 2018;53(4):379-89.

31. Kotake S, Udagawa N, Takahashi N, Matsuzaki K, Itoh K, Ishiyama S, et al. IL-17 in synovial fluids from patients with rheumatoid arthritis is a potent stimulator of osteoclastogenesis. *The Journal of clinical investigation.* 1999;103(9):1345-52.

32. Gravallese EM, and Schett G. Effects of the IL-23-IL-17 pathway on bone in spondyloarthritis. *Nature reviews Rheumatology.* 2018;14(11):631-40.

33. Hersh AO, and Prahalad S. Immunogenetics of juvenile idiopathic arthritis: A comprehensive review. *Journal of autoimmunity.* 2015;64:113-24.

34. Colbert RA. Classification of juvenile spondyloarthritis: Enthesitis-related arthritis and beyond. *Nature reviews Rheumatology.* 2010;6(8):477-85.

35. Roumier A, Olivo-Marin JC, Arpin M, Michel F, Martin M, Mangeat P, et al. The membrane-microfilament linker ezrin is involved in the formation of the immunological synapse and in T cell activation. *Immunity.* 2001;15(5):715-28.

36. Bai RJ, Liu D, Li YS, Tian J, Yu DJ, Li HZ, et al. OPN inhibits autophagy through CD44, integrin and the MAPK pathway in osteoarthritic chondrocytes. *Frontiers in endocrinology.* 2022;13:919366.

37. Clatworthy MR. B-cell regulation and its application to transplantation. *Transplant international : official journal of the European Society for Organ Transplantation.* 2014;27(2):117-28.

38. Su H, Na N, Zhang X, and Zhao Y. The biological function and significance of CD74 in immune diseases. *Inflammation research : official journal of the European Histamine Research Society [et al].* 2017;66(3):209-16.

39. De Silvestri A, Capittini C, Poddighe D, Marseglia GL, Mascaretti L, Bevilacqua E, et al. HLA-DRB1 alleles and juvenile idiopathic arthritis: Diagnostic clues emerging from a meta-analysis. *Autoimmunity reviews.* 2017;16(12):1230-6.

40. Adler LN, Jiang W, Bhamidipati K, Millican M, Macaubas C, Hung SC, et al. The Other Function: Class II-Restricted Antigen Presentation by B Cells. *Frontiers in immunology.* 2017;8:319.

41. Puri KD, Di Paolo JA, and Gold MR. B-cell receptor signaling inhibitors for treatment of autoimmune inflammatory diseases and B-cell malignancies. *International reviews of immunology.* 2013;32(4):397-427.

42. Ma WT, Gao F, Gu K, and Chen DK. The Role of Monocytes and Macrophages in Autoimmune Diseases: A Comprehensive Review. *Frontiers in immunology.* 2019;10:1140.

43. Rana AK, Li Y, Dang Q, and Yang F. Monocytes in rheumatoid arthritis: Circulating precursors of macrophages and osteoclasts and, their heterogeneity and plasticity role in RA pathogenesis. *International immunopharmacology.* 2018;65:348-59.

44. Ożańska A, Szymczak D, and Rybka J. Pattern of human monocyte subpopulations in health and disease. *Scandinavian journal of immunology.* 2020;92(1):e12883.

45. Dinarello CA, Simon A, and van der Meer JW. Treating inflammation by blocking interleukin-1 in a broad spectrum of diseases. *Nature reviews Drug discovery.* 2012;11(8):633-52.

46. Zhang F, Wei K, Slowikowski K, Fonseka CY, Rao DA, Kelly S, et al. Defining inflammatory cell states in rheumatoid arthritis joint synovial tissues by integrating single-cell transcriptomics and mass cytometry. *Nature immunology.* 2019;20(7):928-42.

47. Hirano T. IL-6 in inflammation, autoimmunity and cancer. *International immunology.* 2021;33(3):127-48.

48. Naruke A, Nakano R, Nunomura J, Suwabe Y, Nakano M, Namba S, et al. Tpl2 contributes to IL-1β-induced IL-8 expression via ERK1/2 activation in canine dermal fibroblasts. *PloS one.* 2021;16(11):e0259489.

49. Stansfield BK, and Ingram DA. Clinical significance of monocyte heterogeneity. *Clinical and translational medicine.* 2015;4:5.

50. Hong X, Meng S, Tang D, Wang T, Ding L, Yu H, et al. Single-Cell RNA Sequencing Reveals the Expansion of Cytotoxic CD4(+) T Lymphocytes and a Landscape of Immune Cells in Primary Sjögren's Syndrome. *Frontiers in immunology.* 2020;11:594658.

51. Mandric I, Schwarz T, Majumdar A, Hou K, Briscoe L, Perez R, et al. Optimized design of single-cell RNA sequencing experiments for cell-type-specific eQTL analysis. *Nature communications.* 2020;11(1):5504.

52. Pan L, Dinh HQ, Pawitan Y, and Vu TN. Isoform-level quantification for single-cell RNA sequencing. *Bioinformatics (Oxford, England).* 2022;38(5):1287-94.

53. Butler A, Hoffman P, Smibert P, Papalexi E, and Satija R. Integrating single-cell transcriptomic data across different conditions, technologies, and species. *Nature biotechnology.* 2018;36(5):411-20.

54. McGinnis CS, Murrow LM, and Gartner ZJ. DoubletFinder: Doublet Detection in Single-Cell RNA Sequencing Data Using Artificial Nearest Neighbors. *Cell systems.* 2019;8(4):329-37.e4.

55. Skinnider MA, Squair JW, Kathe C, Anderson MA, Gautier M, Matson KJE, et al. Cell type prioritization in single-cell data. *Nature biotechnology.* 2021;39(1):30-4.

56. Chicco D, and Jurman G. A brief survey of tools for genomic regions enrichment analysis. *Frontiers in bioinformatics.* 2022;2:968327.

57. Qiu X, Mao Q, Tang Y, Wang L, Chawla R, Pliner HA, et al. Reversed graph embedding resolves complex single-cell trajectories. *Nature methods.* 2017;14(10):979-82.

58. Efremova M, Vento-Tormo M, Teichmann SA, and Vento-Tormo R. CellPhoneDB: inferring cell-cell communication from combined expression of multi-subunit ligand-receptor complexes. *Nature protocols.* 2020;15(4):1484-506.

**Figures and figure legends:**


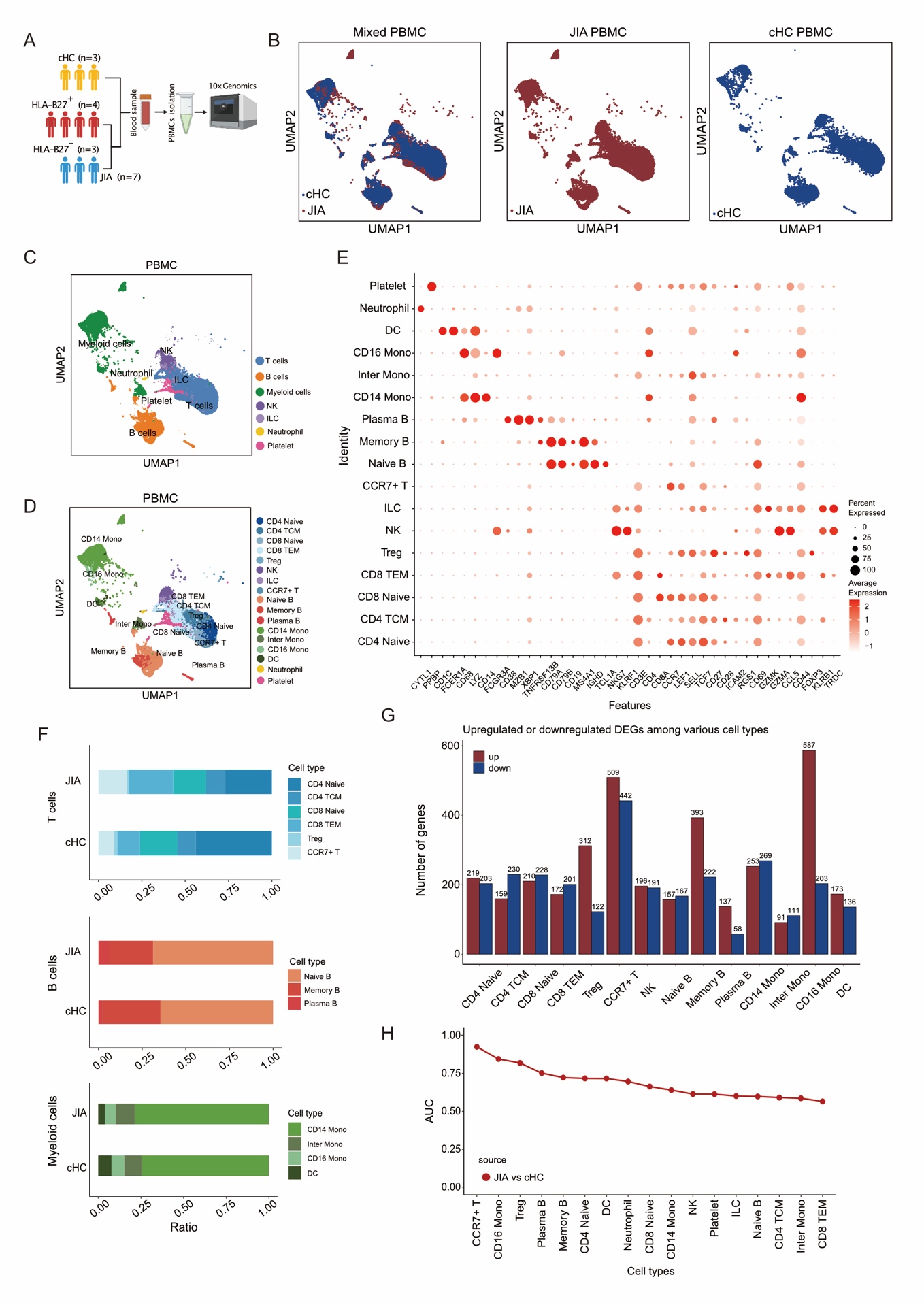


**Figure 1. Immune cells composition differs in peripheral blood from JIA patients and cHC.** **A.** Overview of the research workflow. cHC: children healthy controls; HLA-B27: Human leukocyte antigen B27; PBMCs: Peripheral blood mononuclear cells. **B.** UMAP projection of the JIA and cHC. **C.** Integrated UMAP graph of B cells, T cells, Myeloid cells, NK, ILC, Neutrophil and Platelet derived from our research, colored by cell types. Among the identified immune cells, 39, 615 cells from JIA and 17, 127 cells from cHC. **D.** Integrated UMAP graph of T cell subtypes, B cell subtypes and Myeloid cell subtypes, colored by cell subtypes. **E.** Dot plot showing the expression level of classical cell markers used to assign cell identity. **F.** The proportion of different subtypes of T cells, B cells and Myeloid cells. **G.** Upregulated and downregulated differentially expressed genes (DEGs) in immune cell subtypes of JIA compared to cHC and the corresponding GO enrichment results. **H.** The AUC score of Augur algorithm was used to rank the cell types.


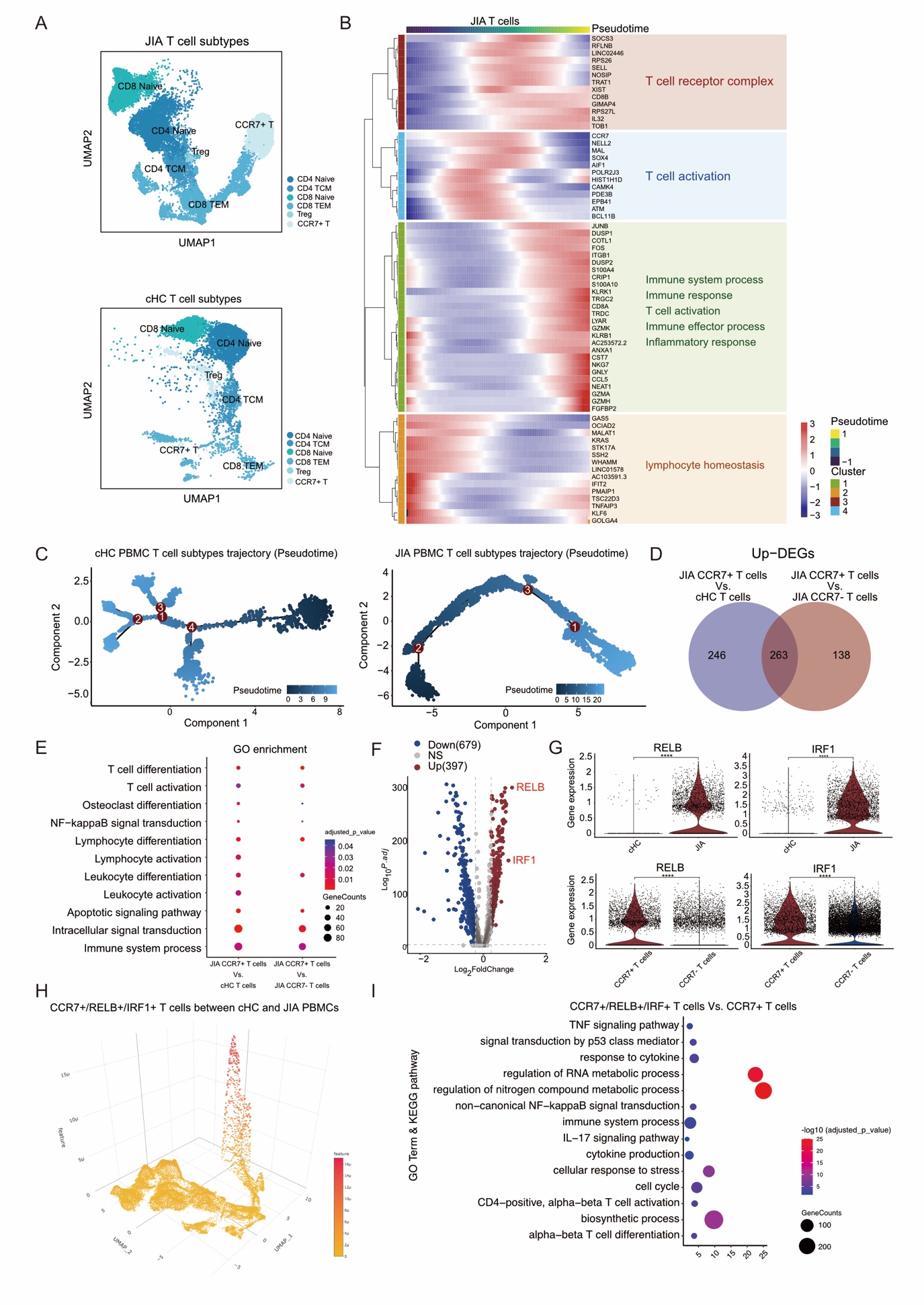


**Figure 2. The proportion of CCR7+/RELB+/IRF1+ triple positive T cells is an important feature of JIA patients.** **A.** Integrated UMAP graph of T cell subtypes in JIA patients and cHC respectively, colored by T cell subtypes. **B.** Heatmap showing dynamic changes in gene expression in T cell subtypes of JIA patients. **C.** Differentiation trajectories of T cell subtypes in JIA patients and cHC, respectively. **D.** Venn plot for the overlap of the upregulated DEGs by CCR7^+^ T cells in JIA patients compared with CCR7^+^ T cells in cHC and other T cells in JIA patients, respectively. **E.** The GO enrichment results of the upregulated DEGs by CCR7^+^ T cells in JIA patients compared with CCR7^+^ T cells in cHC and other T cells in JIA patients, respectively. **F.** Volcano plot showing differentially expressed genes (DEGs) between CCR7^+^ T cells in JIA and CCR7^+^ T cells in cHC. **G.** Violin plots showing the differences in expression levels of *RELB* and *IRF1* between CCR7^+^ T cells in cHC and CCR7^+^ T cells in JIA patients; And the differences in expression levels of *RELB* and *IRF1* between CCR7^+^ T cells in JIA patients and other T cells in JIA patients. **H.** Dispersed expression of *CCR7*, *RELB* and *IRF1* cell populations. **I.** The GO and KEGG enrichment results of the upregulated DEGs by CCR7+/RELB+/IRF1+ triple positive T cells in JIA patients compared with other CCR7^+^ T cells in JIA patients. P values were calculated by Wilcox test. *p < 0.05, **p < 0.01, ***p < 0.001.


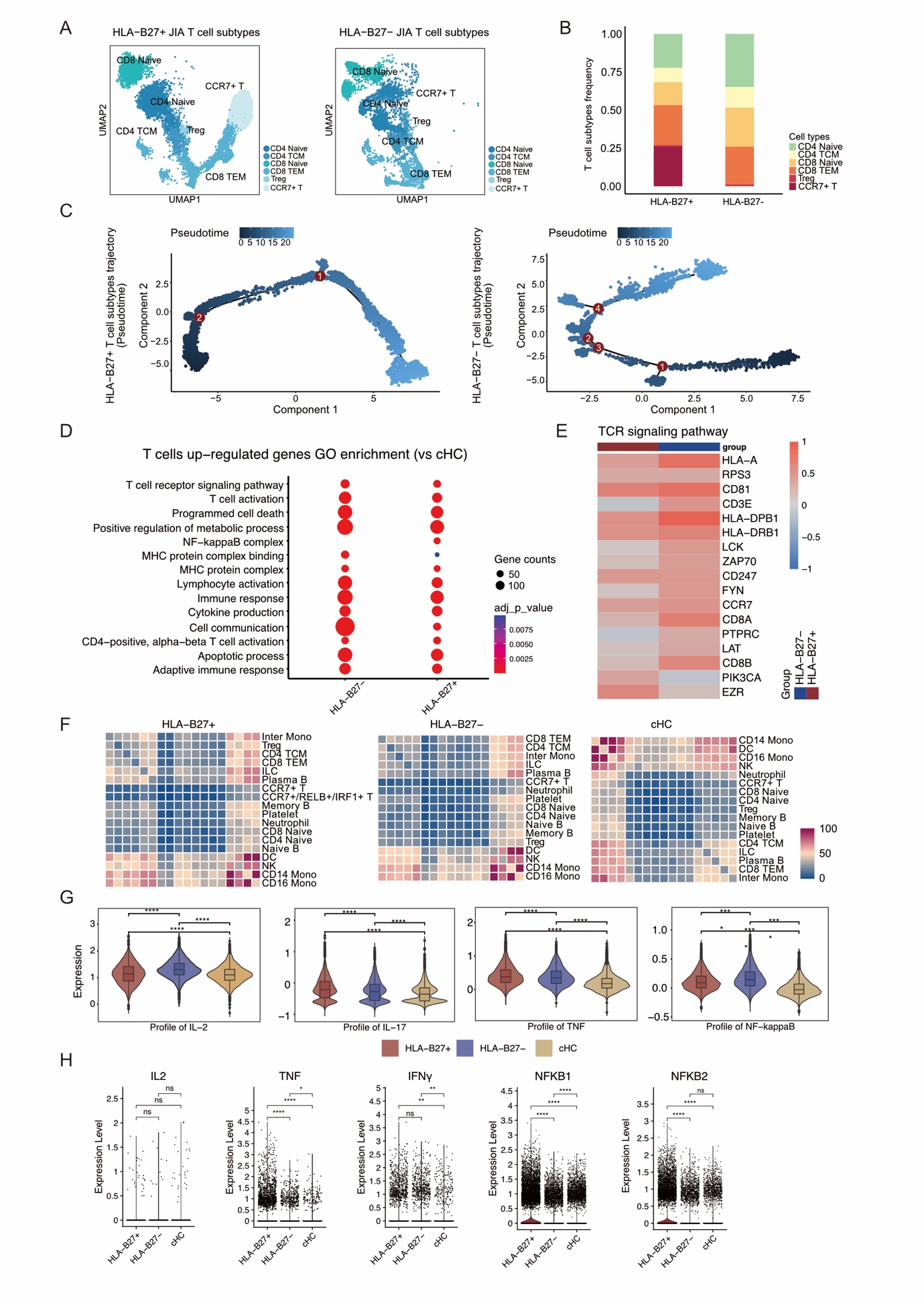


**Figure 3. CCR7+/RELB+/IRF1+ triple positive T cells are mainly present in T cells from HLA-B27^+^ JIA patients.** **A.** Integrated UMAP graph of T cell subtypes in HLA-B27^+^ JIA patients and HLA-B27^-^ JIA patients, colored by T cell subtypes. **B.** The proportion of different subtypes of T cells between HLA-B27^+^ JIA patients and HLA-B27^-^ JIA patients. **C.** Differentiation trajectories of T cell subtypes in HLA-B27^+^ JIA patients and HLA-B27^-^ JIA patients, respectively. **D.** The GO enrichment results of the upregulated DEGs by T cells in HLA-B27^-^ JIA and HLA-B27^+^ JIA compared with cHC, respectively. **E.** Heatmap of the gene expression of TCR signaling pathway in T cells between HLA-B27^-^ JIA patients and HLA-B27^+^ JIA patients. **F.** Heatmaps showing the interaction between immune cells in HLA-B27^+^ JIA, HLA-B27^-^ JIA and cHC, respectively. **G.** Profile scores of IL-2, IL-17, TNF and NF-kappaB in T cells among three groups. **H.** Violin plots showing the differences in expression levels of *IL2*, *TNF*, *IFNγ*, *NFKB1* and *NFKB2* in T cells from HLA-B27^+^ JIA, HLA-B27^-^ JIA and cHC. P values were calculated by Wilcox test. *p < 0.05, **p < 0.01, ***p < 0.001.


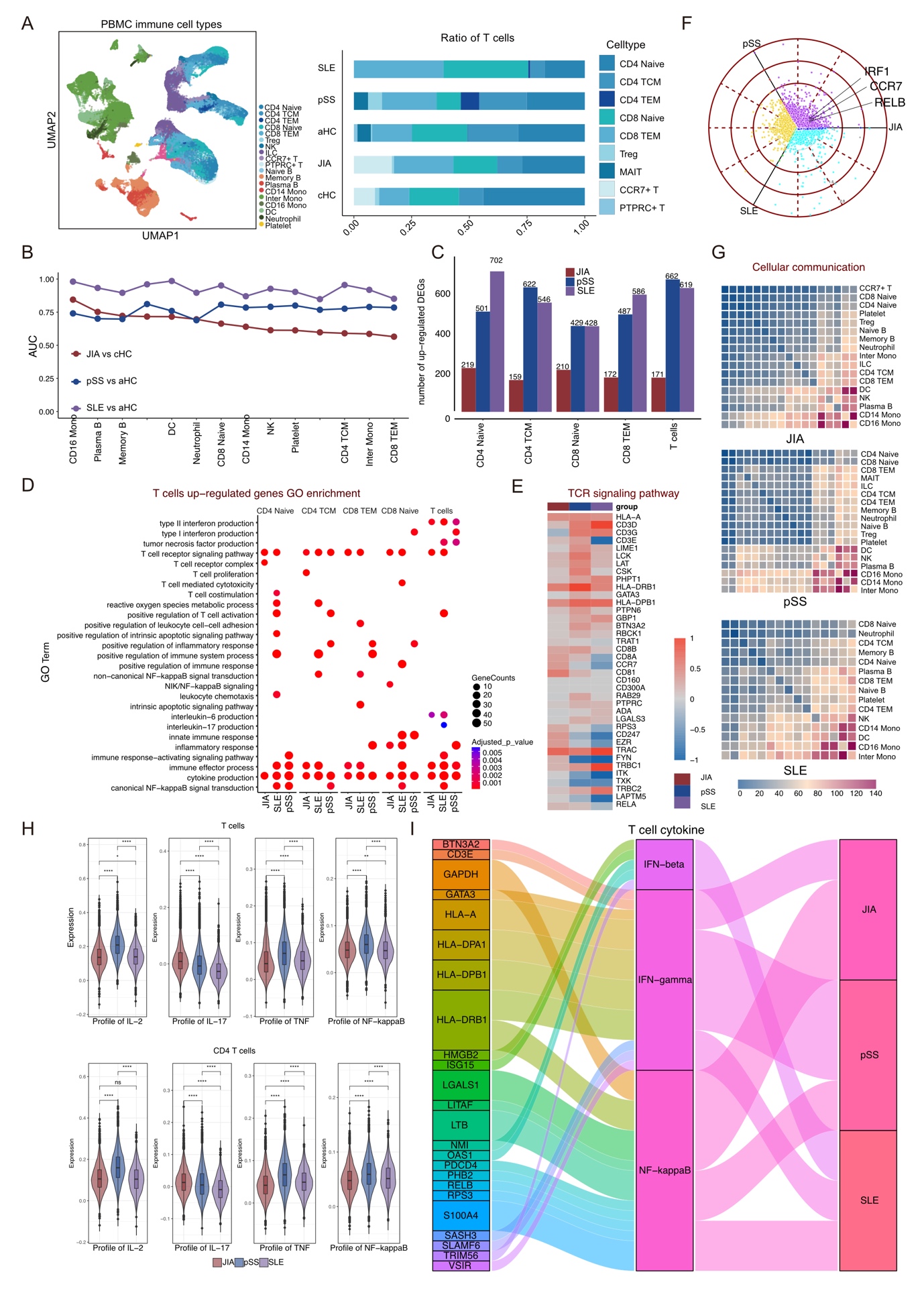


**Figure 4. pSS patients and SLE patients lack T cells that express high levels of RELB and IRF1.** **A.** Integrated UMAP graph of 13,7454 immune cells derived from our study, colored by cell types. Among the identified immune cells, 24,962 cells from pSS, 32,655 cells from SLE and 23,095 cells from aHC. Bar plot showing the proportion of different subtypes of T cells, B cells and Myeloid cells. **B.** Augur algorithm was used to rank the cell types of JIA patients, pSS patients and SLE patients. **C.** Upregulated DEGs in T cell subtypes of JIA patients compared with cHC, and upregulated DEGs in T cell subtypes of pSS patients and SLE patients compared with aHC. **D.** GO enrichment results of upregulated DEGs in T cell subtypes of JIA patients, pSS patients and SLE patients. **E.** Heatmap of the gene expression of TCR signaling pathway of T cells in JIA patients, pSS patients and SLE patients. **F.** Polar plot gene expression across JIA, pSS and SLE for T cells. Off-axis points reflect shared expressions among patients with different autoimmune diseases. **G.** Heatmaps showing the interaction between immune cells in HLA-B27^+^ JIA, HLA-B27^-^ JIA and cHC, respectively. **H.** Profile scores of IL-2, IL-17, TNF and NF-kappaB in T cells and CD4 T cells among three groups. **I.** Sankey diagrams showing the difference in IFN-β, IFN-γ and NF-kappaB of T cells in JIA patients, pSS patients and SLE patients. P values were calculated by Wilcox test. *p < 0.05, **p < 0.01, ***p < 0.001.


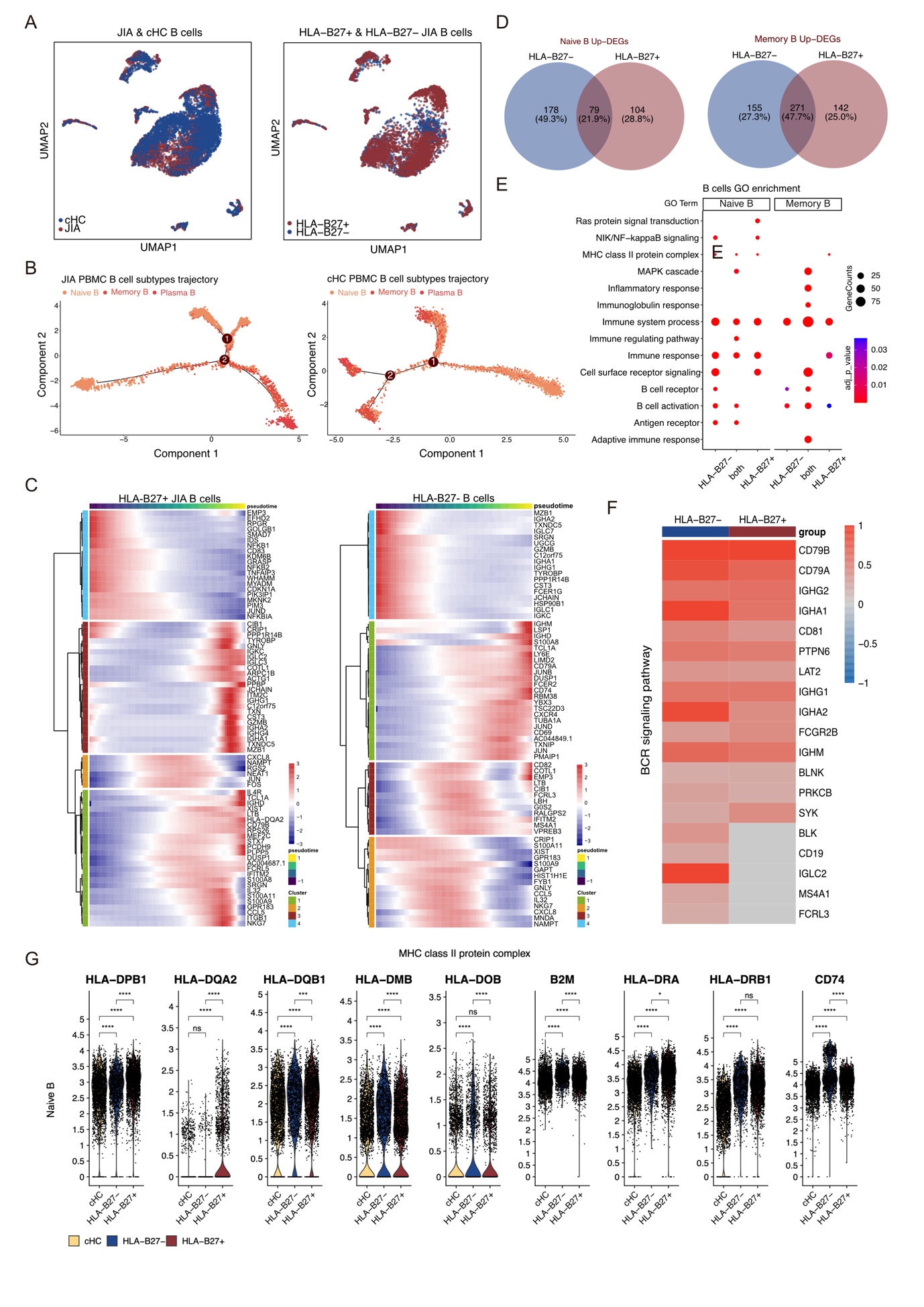


**Figure 5. Different B cell subtypes participate in JIA.** **A.** UMAP projections of B cells from JIA patients and cHC. **B.** Differentiation trajectories of B cell subtypes in JIA patients and cHC, respectively. **C.** Heatmaps showing dynamic changes in gene expression in B cell subtypes of HLA-B27^+^ JIA patients and HLA-B27^-^ JIA patients, respectively. **D.** Venn plots for the overlaps of the upregulated DEGs between HLA-B27^-^ JIA patients and HLA-B27^+^ JIA patients in Naive B and Memory B. **E.** The GO enrichment results of the upregulated genes between HLA-B27^-^ JIA patients and HLA-B27^+^ JIA patients in Naive B and Memory B. **F.** Heatmap of the gene expression of BCR signaling pathway in Memory B between HLA-B27^-^ JIA patients and HLA-B27^+^ JIA patients. **G.** Violin plots showing the differences in gene expression of MHC class II protein complex in PBMC for Naive B of HLA-B27^-^ JIA patients and HLA-B27^+^ JIA patients. P values were calculated by Wilcox test. *p < 0.05, **p < 0.01, ***p < 0.001.


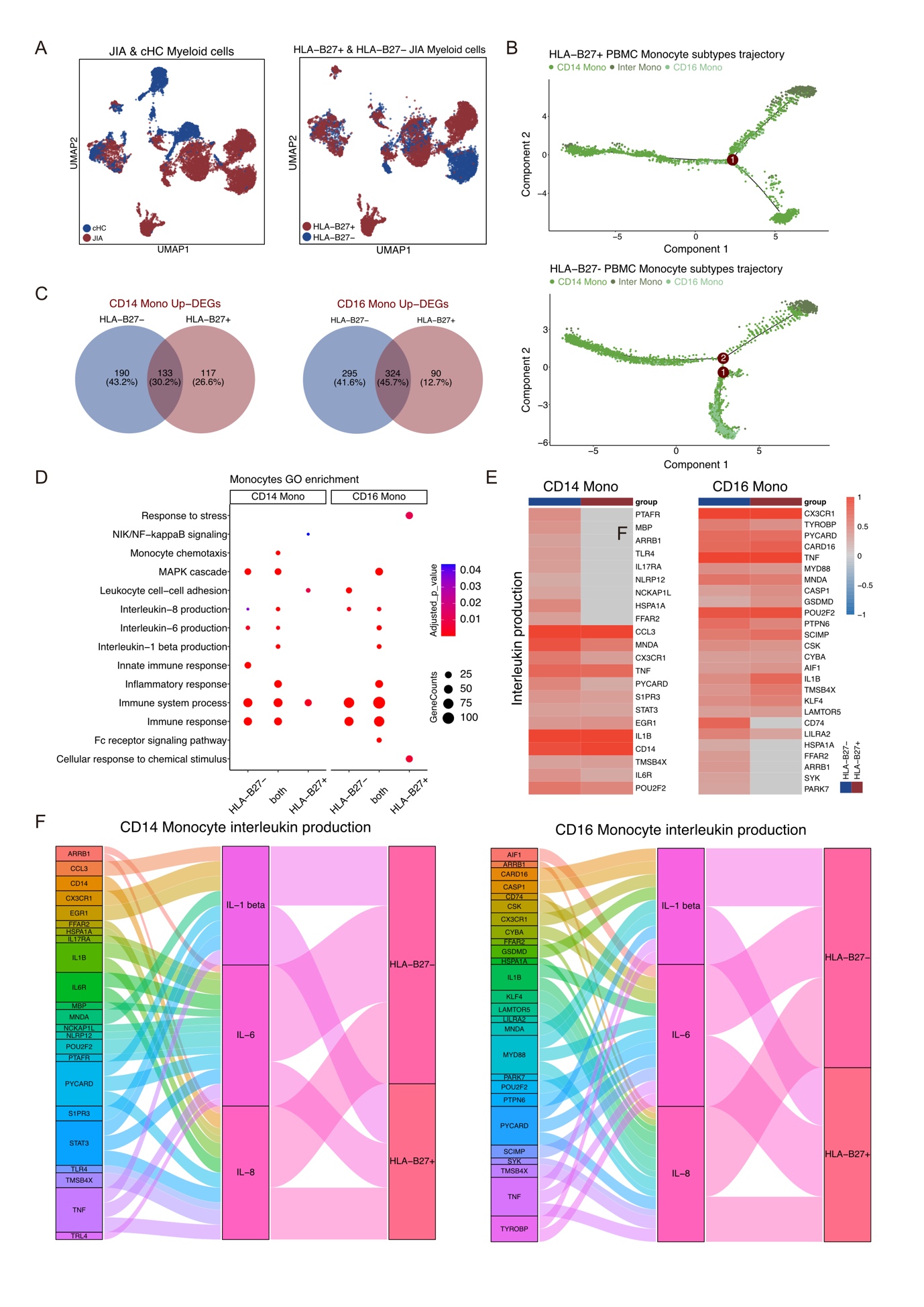


**Figure 6. Different Myeloid cell subtypes participate in JIA.** **A.** UMAP projections of Myeloid cells from JIA patients and cHC. **B.** Differentiation trajectories of Monocyte subtypes in HLA-B27^+^ JIA patients and HLA-B27^-^ JIA patients, respectively. **C.** Venn plots for the overlaps of the upregulated DEGs between HLA-B27^-^ JIA patients and HLA-B27^+^ JIA patients in CD14 Mono and CD16 Mono. **D.** The GO enrichment results of the upregulated DEGs between HLA-B27^-^ JIA patients and HLA-B27^+^ JIA patients in CD14 Mono and CD16 Mono. **E.** Heatmaps of the gene expression of Interleukin production in CD14 Mono and CD16 Mono between HLA-B27^-^ JIA patients and HLA-B27^+^ JIA patients. **F.** Sankey diagrams showing the difference in the pro-inflammatory interleukin between CD14 Mono and CD16 Mono for HLA-B27^-^ JIA patients and HLA-B27^+^ JIA patients.
